# Pervasive under-dominance in gene expression underlying emergent growth trajectories in *Arabidopsis thaliana* hybrids

**DOI:** 10.1186/s13059-023-03043-3

**Published:** 2023-09-04

**Authors:** Wei Yuan, Fiona Beitel, Thanvi Srikant, Ilja Bezrukov, Sabine Schäfer, Robin Kraft, Detlef Weigel

**Affiliations:** Department of Molecular Biology, Max Planck Institute for Biology Tübingen, 72076 Tübingen, Germany

## Abstract

**Background:**

Complex traits, such as growth and fitness, are typically controlled by a very large number of variants, which can interact in both additive and non-additive fashion. In an attempt to gauge the relative importance of both types of genetic interactions, we turn to hybrids, which provide a facile means for creating many novel allele combinations.

**Results:**

We focus on the interaction between alleles of the same locus, i.e., dominance, and perform a transcriptomic study involving 141 random crosses between different accessions of the plant model species *Arabidopsis thaliana*. Additivity is rare, consistently observed for only about 300 genes enriched for roles in stress response and cell death. Regulatory rare-allele burden affects the expression level of these genes but does not correlate with F_1_ rosette size. Non-additive, dominant gene expression in F_1_ hybrids is much more common, with the vast majority of genes (over 90%) being expressed below the parental average. Unlike in the additive genes, regulatory rare-allele burden in the dominant gene set is strongly correlated with F_1_ rosette size, even though it only mildly covaries with the expression level of these genes.

**Conclusions:**

Our study underscores under-dominance as the predominant gene action associated with emergence of rosette growth trajectories in the *A. thaliana* hybrid model. Our work lays the foundation for understanding molecular mechanisms and evolutionary forces that lead to dominance complementation of rare regulatory alleles.

**Supplementary Information:**

The online version contains supplementary material available at 10.1186/s13059-023-03043-3.

## Background

When expression and inheritance of a trait is under the control of many genes, it is considered a quantitative or complex trait, with growth- and fitness-related traits being almost always complex traits [1, 2]. The complexity of these traits comes not only from the large number of underlying genes/loci, but also from the multitude of potential allelic interactions within and between the genes involved. The best-understood type of allelic interaction is the additive effect, in which different alleles contribute to a trait co-dominantly, and the offspring have an intermediate trait value that is close to the average of the two parental alleles. By definition, non-additive genetic effects are any deviations from this additive scenario, with two common examples being dominance and epistasis [1]. Due to limitations in technical and statistical frameworks, non-additive effects are much less studied and understood than additive effects [3]. Nevertheless, there is evidence that non-additive effects can be pervasive and contribute substantially to what had for some time been perceived as “missing-heritability” [4–6]. Hybrids provide a unique opportunity to study the dominant portion of non-additive effects, enabling direct comparison of the relative importance of additivity and dominance to phenotypes of interest.

Causal genetic variants often exert their functional effects by modulating gene expression. Measuring gene expression differences and associating the variation with complex traits provides information regarding biological functions and processes causing natural phenotypic variation [7]. Over the last decade, statistical frameworks such as transcriptome-wide association (TWA) and expression QTL (eQTL) have rapidly matured, providing insights into molecular functions of complex traits [7, 8]. The highly quantitative nature of transcriptomic, especially RNA-seq data provides an excellent opportunity for tracking additive versus dominant gene actions. By comparing the expression level of a gene in F_1_ heterozygotes to that of the parental average, one can easily calculate the degree of its non-additivity [9].

Above-ground biomass accumulation is a fitness-related trait that has an important bearing on both the local adaptation of wild plants [10] and the performance of agricultural species [11, 12]. In the inbreeding plant *Arabidopsis thaliana*, rosette size, a close proxy for above-ground biomass, is not only a primary indicator of growth and general performance [13, 14], but also both highly variable [14, 15] and strongly associated with fitness [15, 16]. The range of variation in this trait can be substantially increased by including F_1_ hybrids. While rare combinations are smaller than any naturally occurring accession [17, 18], most F_1_ hybrids have larger rosettes [19–21], an emergent positive phenotype that in outbreeding crops is usually termed heterosis. F_1_ hybrids are particularly interesting systems to study, as alleles that are naturally segregated into different genomes are brought into contact with each other, leading to numerous novel genetic interactions [22, 23]. It has long been postulated that these novel genetic interactions, both additive and non-additive, may contribute to hybrid performance [22, 24, 25].

We are interested in understanding how common additive and non-additive gene action is, and how it relates, if at all, to growth phenotypes in F_1_ hybrids. We focus on dominance, though both dominance and epistasis have been hypothesized to be critical for hybrid phenotypes [9]. Specifically, we would like to learn at the species level (i) whether additive and dominant gene actions occur at a similar frequency, (ii) whether the gene actions are mostly specific to parental combinations, or if certain genes and pathways particularly frequently exhibit one of the effects, (iii) and whether emergent phenotypes in F_1_ hybrids are more likely to result from additive or dominant gene actions. *Arabidopsis thaliana* provides a powerful system to address these questions, due to the wealth of genomic resources and large collection of natural accessions [26]. Previous studies of intra-specific *A.* *thalian* hybrids have provided insights into mechanisms affecting hybrid performance, such as the mitigation of defense-growth tradeoffs in superior hybrids [27], and on the flip side, greatly compromised growth in hybrids due to incompatible allelic interaction and excessive activation of defense [17].

We designed a study in *A. thaliana* that surveyed not only a broad range of the species’ genetic diversity, but also allowed for the detection of interactions between an exceptionally large number of alleles. We find non-additivity in gene expression in F_1_ hybrids to be common, with dominant genes being much more commonly expressed below the parental average (mid-parent value, MPV) than above it. Expression close to the MPV in turn is rare, with a substantial fraction of such genes having a role in biotic defense pathways, suggesting that defense is particularly well buffered.

## Results

### Dominant gene action is more abundant in F_1_ hybrids

**Fig. 1:**
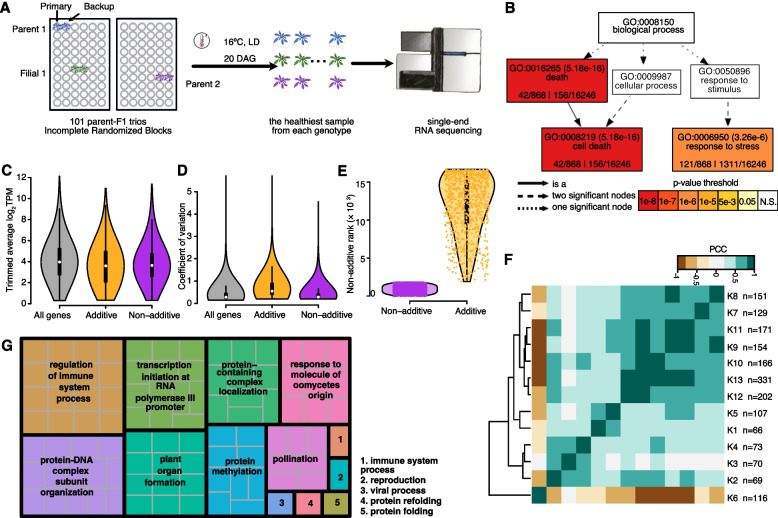
Summary of additive and dominant genes. **A** Experimental setup. Note that not all trios were completely sequenced and analyzed. **B** GO-term (biological process) enrichment of additive genes. **C**, **D** Both additive and dominant genes showed average transcript abundance (**C**) and coefficient of variation (**D**) profiles comparable to those of all genes in the background. **E** Additive genes ranked low by their dominance score. **F** Correlation among all dominant gene clusters. Pearson correlation coefficients were calculated using cluster average. **G** Revigo summary of biological processes enriched in dominant genes

For our work, we drew on resources from the 1001 Genomes Project for this species [26], crossing re-sequenced, naturally inbred accessions to generate a panel of F_1_ hybrids. To broadly survey possible genetic interactions, and to evaluate whether consistent patterns of additive and dominant gene expression exist, we carried out an RNA-seq experiment for which 101 parent-F_1_ trios, i.e., each F_1_ hybrid and their inbred parents, were planted (Fig. 1A, Additional file 2: Table S1, “Methods” section). Of all genotypes, 82 F_1_s and 124 inbred parents were included in the final analyses. Whole-rosette sizes, a good proxy for biomass (Additional file 1: Fig. S1, “Methods” section), were measured in inbreds and F_1_s.

The expression of many genes changes in F_1_ hybrids relative to the parents [9]. Under an additive model, gene expression in F_1_ hybrids is close to the parental average. We asked whether there are genes that are almost always additively, or dominantly expressed in F_1_s across all trios. Out of 16,667 expressed genes that passed our filter (Methods), we identified close to 900 genes that were consistently expressed in an additive fashion (Additional file 1: Fig. S2, “Methods” section), and 1,805 genes (Additional file 3: Table S2) that were consistently expressed in a dominant fashion (Additional file 1: Fig. S3, “Methods” section) in all trios. Both the additive and the dominant genes had distribution profiles of transcript abundances and coefficients of variation that were similar to background genes (Fig. 1C, D), i.e., including both high- and low-abundance transcripts as well as ones that varied little across samples or ones that varied substantially. This indicates that expression level and variance did not greatly bias our ability to discover specific gene expression patterns. Parenthetically, the great majority of the additive genes had very low ranks on their dominance score (Fig. 1E), confirming that our gene calling algorithm was successfully selected for contrasting gene actions. Within our dominant genes, only 150 (~ 8%) of these were consistently expressed above MPV in the F_1_s. For the great majority of genes, the low expression level was dominant, such that most genes were expressed in the F_1_s at a level between the lower parent and the MPV.

Gene Ontology (GO) term analysis revealed that the additive genes are strongly enriched in cell death- and stress response-related processes (Fig. 1B). If a gene is typically expressed close to the parental average, then the expression values in the parents will be more broadly distributed than in the hybrids, because extreme parents would usually have been crossed to less extreme parents. We therefore interpret our result as indicating that the cell death- and stress-response pathways are systematically buffered in F_1_s. In comparison, dominant genes are featured in much more diverse biological processes (Fig. 1G), including regulation of immune system process, ribosomal RNA transcription, plant organ formation, and others.

Dominant gene expression is thus pervasive in F_1_ hybrids, with the overwhelming majority of genes expressed below the parental average. Many biological processes are affected in the F_1_s by expression dominance, as the GO enrichment suggested.

### Dominant genes covary with size

We reduced the dimensionality of our dominant gene set by grouping genes with a similar behavior across samples via k-means clustering (*k* = 13). We examined how well the behavior of different clusters across samples was correlated (Fig. 1F), finding that one particular cluster (cluster 6, *n* = 116) behaved in a manner that was opposite to that of all other clusters.

**Fig. 2:**
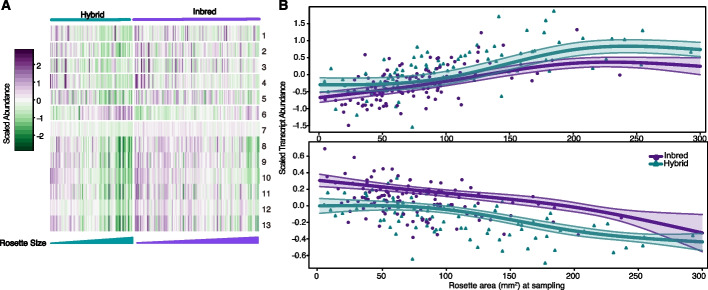
Dominant gene expression level correlates with final rosette size. **A** Heatmap showing the average expression of each dominant gene cluster (K-means) in each sample, sorted into F_1_s and inbred parental lines, and arranged by ascending final rosette size. Clusters 1–5 showed no clear expression-size association. Cluster 6 (*n* = 107) showed a positive association, and clusters 7–13 (*n* = 151) showed a negative association **B** Linear-mixed-model spline fitting of exemplary clusters. Top: cluster 6, which showed positive expression-size association; bottom: cluster 7, which showed negative expression-size association; points: cluster mean expression in each sample; shaded area: 95% Bayesian credible intervals. The systematic differences in expression levels across the entire rosette-size range seen in F_1_ hybrids are consistent with F_1_s being larger than parents

Probing into the underlying commonalities between the genes that drove the clustering, we discovered that the mean expression value for eight of the clusters (clusters 6–13) covaried with rosette size in both inbreds and hybrids. The most distinct cluster 6 showed a clear positive correlation, while the other clusters were negatively correlated with rosette size (Fig. 2A). While the general trend of correlation between gene expression and rosette size remained the same in F_1_s and the inbred parental lines, the shape of the correlation differed.

To obtain further insight into the above observations, we formally investigated the relationship between the average gene expression of each cluster and rosette size, by performing Bayesian linear-mixed-model spline fitting (Fig. 2B, Additional file 1: Fig. S4, “Methods” section). Clusters 1–5 showed no clear trend of association between expression and size, cluster 6 showed expression increasing in parallel with rosette size (Fig. 2B, top panel), and the remaining clusters showed expression decreasing with increasing rosette size. For these seven clusters, F_1_ hybrids tended to have lower expression than the inbreds across the entire range of rosette size (Fig. 2B, bottom panel). GO analysis did not indicate that clusters were specific for particular biological processes.

Therefore, a large number (*n* = 1420) of dominant genes showed covariation of expression level with rosette size. F_1_ hybrids systematically exhibited a shift towards either lower or higher expression levels in the direction consistent with the change in rosette size relative to inbred parental lines. The trend is unique for dominant genes, as repetitive random subsets of the transcriptome showed neither profound covariation with size nor a systematic shift in the expression level in F_1_s (Additional file 1: Fig. S5).

### F1 exhibits robust growth advantage

**Fig. 3:**
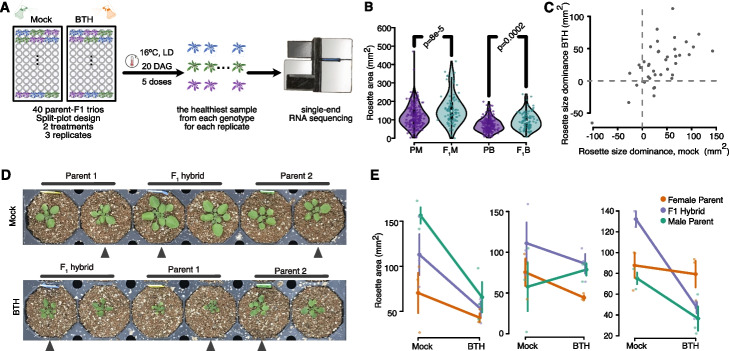
BTH treatment reduced rosette size in both inbreds and F_1_s. **A** Experimental design. **B** F_1_s maintained a robust growth advantage despite the reduction in rosette size upon BTH treatment. PM: parent mock, F_1_M: F_1_ hybrid mock, PB: parent BTH treated, F_1_B: F_1_ BTH treated. **C** Positive correlation between rosette size dominance under mock and BTH conditions. Numbered labels indicate the ID of the SHB2 trios. **D** Typical rosette phenotype of a trio. **E** Diverse response of three example trios to BTH treatment. Reaction norm lines connect the mean ± SD rosette area of each genotype under both treatments

That gene expression exhibited a systematic shift in the F_1_ hybrids, and that dominant gene expression in the F_1_s covaried with rosette size prompted us to ask (i) whether the degree of dominant expression within individual parent-hybrid trios correlated with rosette size differences between the F_1_s and their parents, and (ii) whether a global perturbation to the plant’s developmental program would affect the F_1_s and the inbreds differently. To this end, we conducted a second experiment in which we applied BTH (acibenzolar-S-methyl), an analog of the defense hormone salicylic acid (SA) (Fig. 3A, “Methods” section), to 40 parent-F_1_ trios. Induction of pathogen defense was chosen as treatment because it causes morphological changes and at least sometimes extensive transcriptional reprogramming, as has been observed in some F_1_ hybrids of *A. thaliana* [28].

F_1_ hybrids are on average considerably larger than the inbred parents (inbred mock: 118.2 ± 61.1 mm^2^, F_1_ mock: 157.1 ± 79.6 mm^2^, inbred BTH: 71.6 ± 35.5 mm^2^, F_1_ BTH: 94.3 ± 44.3 mm^2^, Fig. 3B), consistent with our earlier experiment (Additional file 1: Fig. S6). Neither the distribution of the rosettes of the parents nor those of the F_1_ plants was normal, with the F_1_ population having a significantly higher number of larger individuals (*p* = 8e − 5, two-tailed Kolmogorov–Smirnov).

In a comparison of randomly chosen trios, the F_1_ was almost twice as likely as one of the inbreds to be the larger individual (Cliff’s delta = 0.31). BTH treatment significantly reduced plant size in both inbreds and hybrids (mock: 79.2 ± 3.0 mm^2^, BTH: 39.5 ± 4.2 mm^2^, one-way ANOVA, *p* < 2 × 10^−16^, Fig. 3B, D, Additional file 1: Fig. S7), and induced considerable variation in growth responses (Fig. 3E, Additional file 1: Fig. S8). F_1_s exhibited stronger size reduction but remained to be more likely to be larger than either parent (*p* = 0.0002, two-tailed Kolmogorov–Smirnov, Cliff’s delta = 0.31). Most trios showed similar patterns in rosette size growth emergence after both mock and BTH treatment, with the majority of F_1_s remaining larger than the MPV (Fig. 3C).

Growth advantage is therefore prevalent in the F_1_ hybrids included in both of our experiments (Fig. 3B, Additional file 1: Fig. S6), and is to a great extent robust to a perturbation of the developmental program. Although we cannot rule out that even stronger BTH treatment would eventually render hybrids smaller than the inbreds, it is, however, unlikely to occur in a natural setting, as our treatment already resulted in extremely dwarfed plants.

### Degree of non-additivity correlates with F_1_ growth advantage

**Fig. 4:**
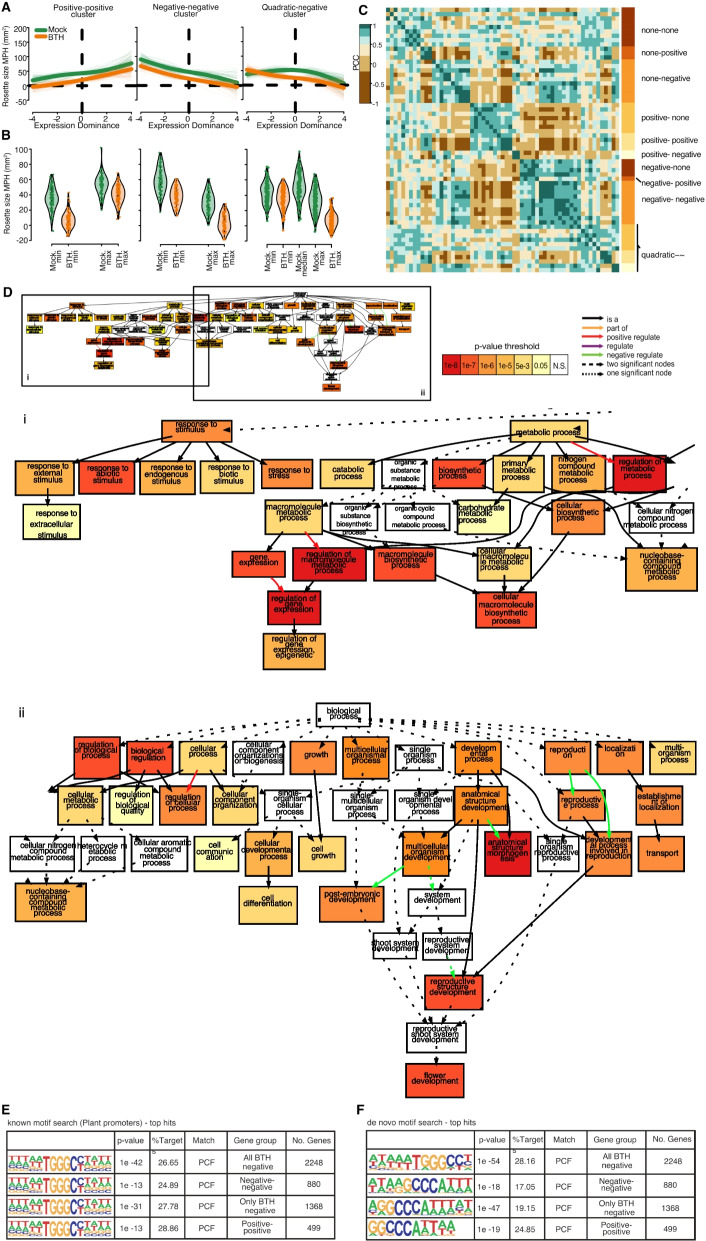
Genes whose degree of expression dominance in trios correlates with hybrid performance. **A** Exemplary clusters of “positive-positive” (left), “negative-negative” (middle), and “quadratic-negative” (right) genes. Thick solid line: spline fitting of the cluster means; thin lines: spline fitting of individual cluster members. **B** Average biomass MPH (the amount that F_1_ rosette size differs from that of corresponding parental mean) for rosette samples with low- vs. high- expression of genes in the same clusters as in A. Each violin depicts the distribution of cluster gene expression averaged across the top and the bottom (and the middle for the quadratic relationship) deciles of samples. **C** Pearson correlation coefficients (PCC) of all 61 clusters based on LMM-spline modeling. The clusters are further sorted into 12 classes labeled on the right according to the relationship between gene expression and biomass under mock or BTH treatment. **D** GO enrichment for genes from the negative cluster. The small plot shows the overall GO network structure, and the positional relationship of the two enlarged graphs of the sub-network (i and ii). **E** Regulatory regions of genes from both positive and negative clusters are enriched for a PCF binding motif. **F** De novo motif search confirmed enrichment of the PCF motif

Having established that dominantly expressed genes are systematically associated with plant size and that F_1_ rosettes frequently exhibit positive size emergence, we investigated whether the degree of expression non-additivity in F_1_s may be associated with this phenotypic non-additivity. We focused on genes showing general response to BTH treatment (*n* = 6371) and asked whether deviations of F_1_ expression values from the MPV of any gene in individual trios exhibited a correlation to the non-additivity in F_1_ rosette size. Clear correlations could be observed for many genes, which could be broadly categorized into (monotonic) positive, (monotonic) negative, or quadratic (Fig. 4A), while in some cases no correlation was observed. We defined 61 groups of genes that fell into these different categories by k-means clustering. To establish the significance of the size-expression correlation, we performed a Wilcoxon signed-rank test on the genes in each of the 61 clusters (Fig. 4B, “Methods” section, Additional file 4: Table S3). Some clusters shared similar relationships between the non-additivity in gene expression and rosette size, therefore we sorted the clusters further into 12 classes reflecting the pattern of correlation under mock and BTH treatment (e.g., “negative-negative” means negative correlation under both treatments, Fig. 4C, “Methods” section). Correlation often changed in response to treatment, with the majority of genes exhibiting a negative correlation with rosette size non-additivity under at least one condition, a trend that increased after BTH treatment (Additional file 1: Fig. S9). This observation is consistent with your finding that negative dominance is more pervasively associated with rosette growth in *A. thaliana*.

The genes with negative size-expression correlation after BTH treatment (Additional file 5: Table S4, “negative genes” hereafter) are enriched for GO terms “regulation of gene expression”, “floral organ development”, and “response to (abiotic) stimuli” (Fig. 4D). Genes with positive correlation under both treatments (Additional file 6: Table S5 “positive genes” hereafter) were moderately enriched for photosynthesis (Additional file 1: Fig. S10). While it seems unlikely, we cannot exclude that the enrichment for abiotic response is due to our analytic focus on BTH-responsive genes, though a parallel GO enrichment test for all BTH-responsive genes did not return significant hits in any biological process. Together these results suggest that the F_1_ transcriptome is systematically repressed in reproductive growth and (abiotic) stress response functions while activated in photosynthesis. Such transcriptome signature of dominance is associated with the rosette growth advantage of the F_1_s, which is pervasive in our system.

To begin to discover potential regulatory mechanisms, we performed motif enrichment analysis among the heterosis-associated genes (“Methods” section). Proliferating cell factor (PCF) and c-Myc transcription factor binding motifs are highly enriched in the promoters of negative genes (569/2248 genes, *p* = 10^−42^, and 433/2248 genes, *p* = 10^−21^, Fig. 4E, Additional file 1: Fig. S11). PCF-binding motifs are also highly enriched in the positive genes (144/499 genes, *p* = 10^−13^, Fig. 4E). These findings were corroborated by de novo motif searches (Fig. 4F). PCF/TCP proteins constitute a conserved plant-specific transcription factor family that includes several regulators of cell cycle, growth, and disease resistance [29, 30]. That both positive and negative genes were enriched for PCF motifs points to these factors as a potential central toggle for global re-modeling of hybrid transcriptomes.

Parenthetically, close to 900 additively expressed genes were called from the second experiment, and 300 of which (hereafter, common additive genes) overlapped with the first experiment (Additional file 1: Fig. S12, Additional file 7: Table S6, “Methods” section). GO-term analysis of the common additive genes revealed enrichment in cell death- and stress response-related processes (Additional file 1: Fig. S12), again confirming that genes from the second experiment were more enriched for defense response than the genes from the first experiment without BTH treatment (Additional file 1: Fig. S13).

### Dominant, not additive complementation underlies the emergence of F_1_ growth advantage

We next asked whether the association between expression level and F_1_ growth has a common genetic basis. Hybrids offer a genomic playground where deleterious alleles from each of the parental genomes may be complemented, one of the leading hypotheses for growth advantage in hybrids. Deleterious alleles are expected to be segregating at lower frequency in the population, and those residing in the gene regulatory regions have been associated with mis-expression of genes in *cis* [31]. To gauge the effect of genetic complementation in the F_1_s, we turned to regulatory rare alleles, looking into the relationship between gene expression level and the average number of regulatory rare SNPs (within 1 kb upstream of genes), which are more likely to have a deleterious effect on gene expression than common SNPs, in both inbred parents and the F_1_s (Methods).

**Fig. 5:**
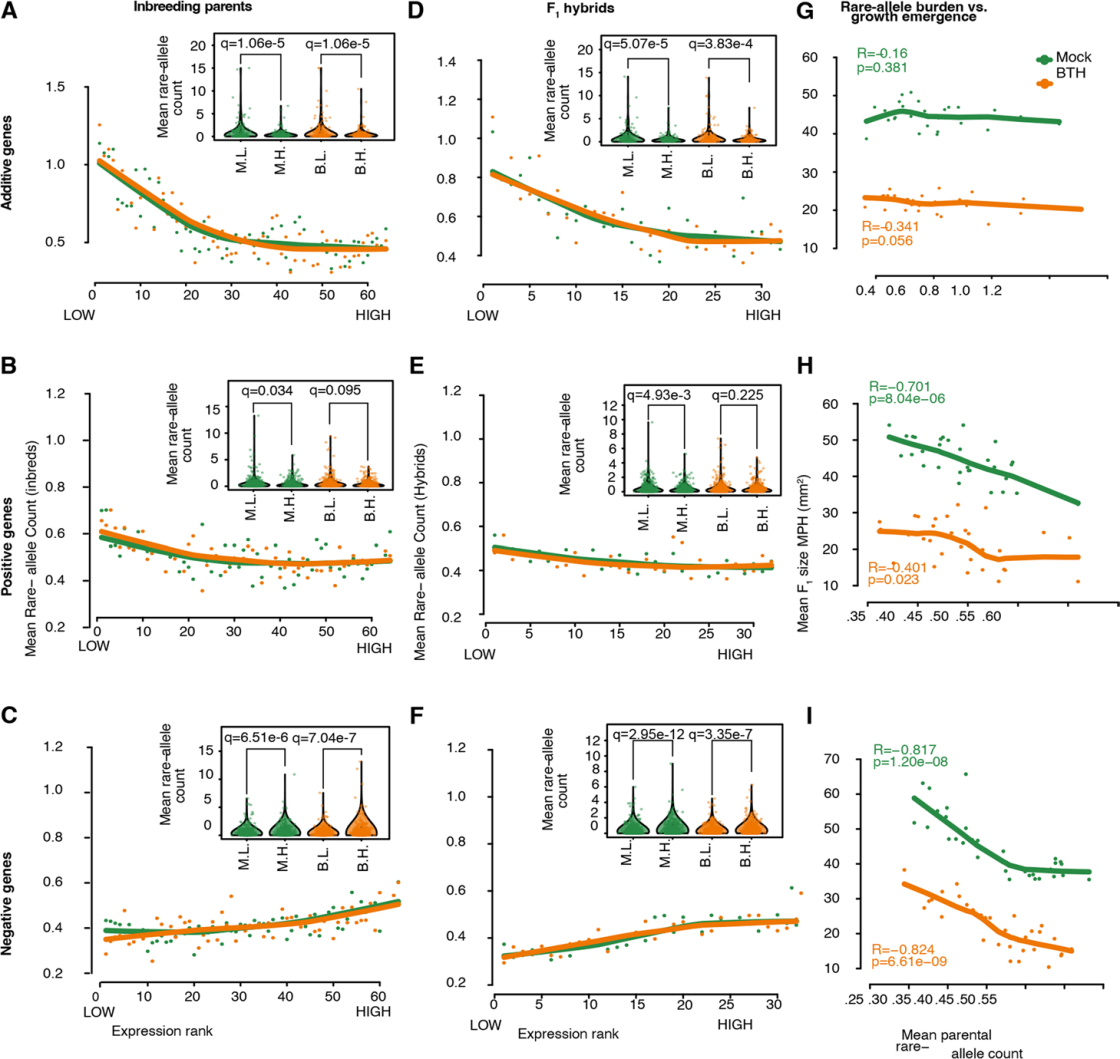
Rare allele burden affects gene expression and the emergence of F_1_ growth advantage. **A**-**C** Association between gene expression rank and upstream rare allele count of additive genes (**A**), positive genes (**B**), and negative genes in inbred parents (**C**). Average upstream rare allele counts were calculated *sensu* Kremling et al. (2018) [31]: for each gene within the gene list (the cluster), all inbred samples received a rank based on their expression value. Across the gene list, average upstream rare allele counts of all samples sharing the same rank were plotted as points, and lines indicate LOWESS trend lines. Insets show the upstream rare-allele count of samples in the top (Mock 10, BTH10) and bottom decile (Mock 1, BTH 1) of expression ranks. **D**–**F** Association between gene expression rank and upstream rare allele count of three gene lists in F_1_ hybrids. F_1_ samples are ranked by expression value the same way as the inbreds in **A**–**C**. The rare allele count for the F_1_s is calculated as the average number of rare alleles between their corresponding parents. **G**–**I** Association between rosette growth emergence and mean parental rare allele burden in additive (**G**), positive (**H**), and negative (**I**) genes. For each gene, F_1_s were ranked by the average number of rare alleles in their parents. Points: average non-additivity in rosette size of all F_1_s sharing the same rank; lines: LOWESS trend lines

Regardless of the treatment, we observed on average a significantly higher rare allele counts upstream of the common additive genes associated with low expression ranks in inbred parents (Fig. 5A, Wilcoxon signed rank-sum test with Benjamin-Hochberg FDR: mock, *α* = 1.1 × 10^−5^, BTH: *α* = 1.1 × 10^−5^). The trend was moderate in the positive genes (Fig. 5B, mock: *α* = 0.03, BTH: *α* = 0.10) and in the opposite direction in the negative genes (Fig. 5C, mock: *α* = 7 × 10^−6^, BTH: *α* = 7 × 10^−7^). Trends in F_1_s were in all three gene groups consistent with what was seen in the parents (Fig. 5D, F, *α* = 5.1 × 10^−5^, additive-mock; *α* = 3.8 × 10^−4^, additive-BTH; *α* = 0.005, positive-mock; *α* = 0.23, positive BTH; *α* = 3 × 10^−12^, negative-mock; *α* =  × 10^−7^, negative-BTH). Note that for the dominantly expressed genes, increased upstream rare-allele count was always associated with an expression pattern that is consistent with a smaller plant. Upstream rare-allele burden therefore tends to lead to more extreme, and deleterious expression of these genes, in parents and the hybrids alike.

We next asked what the likely phenotypic consequence of the upstream rare-allele burden might be. Because dominance in gene expression varied less with rare-allele burden than additive gene expression (Fig. 5A, D), we expected these additive genes to be critical for F_1_ growth advantage. To our surprise, the number of rare alleles upstream of these common additive genes did not seem to be particularly relevant for rosette growth advantage in the F_1_s (Fig. 5G, Pearson correlation *R* = -0.16, *p* = 0.38). Additive complementation may therefore be an inherent property of hybrids without directly influencing growth advantage. In contrast, the number of mean upstream rare alleles in both positive (Pearson correlation *R* = -0.701, *p* = 8.04e − 6) and negative (Pearson correlation *R* = -0.817, *p* = 1.20e − 8) genes is a strong negative predictor of rosette growth advantage in F_1_ hybrids (Fig. 5H, I, suggesting that growth advantage tends to be greater in hybrids derived from parents with fewer rare alleles in the upstream regions of these dominantly expressed genes. We conclude that dominant complementation contributes heavily to the F_1_ growth advantage observed in our system.

## Discussion

Most, if not all, of the growth and survival phenotypes of an organism are complex traits. Although the infinitesimal model predicts that dominance contributes only minimally to most structural traits [32], their impact is expected to be far greater in fitness-related traits [33]. To date, most of the methods for mapping and genomic selection are based on additive effects only [3]. However, incorporating non-additive, i.e. dominant and epistatic, effects into quantitative genetics modeling can improve heritability estimates and accuracy of genomic prediction [34–37]. Limited empirical data exists for non-additive variance estimates, which varies greatly between organisms and can range from around 3 to 15% of total phenotypic variance in humans and animals [38, 39] to a third or more in plants [37, 40, 41]. Adequate knowledge of non-additive genetic action is therefore of pivotal importance for a thorough understanding of the genetic architecture of complex traits, especially those that are fitness-related, and their evolutionary trajectory.

Motivated to better understand the prevalence and consequence of non-additive, especially dominant genetic action, we systematically compared the additivity and dominance in gene expression in *A. thaliana* F_1_ hybrids. Consistent with findings in other systems [24, 42], we found non-additivity to be prevalent. Moreover, there was a prominent association between non-additivity and biomass on a cross-population scale, lending support to the theory of directional dominance being an underlying factor of emergent phenotypes in hybrids [1].

We observed a common occurrence of rosette growth advantage in our F_1_ collection, a metric for heterosis in *A. thaliana*. Previous inferences from smaller sets of *A. thaliana* F_1_ hybrids [19, 20] have suggested similarly. Despite extensive exploitation in breeding and agriculture, we are only beginning to understand the molecular basis and mechanisms behind heterosis due to its highly heterogeneous nature. One recurring question is whether true heterozygote advantage exists due to overdominance at a few loci, or whether there is merely additive complementation of numerous mildly unfavorable recessive alleles [43]. The search for overdominant loci has proven to be exceedingly difficult, due to the genetic load of (domesticated) species as well as limitations in genetic mapping [44], which cannot be overcome with modern marker technology. In tomato and rice, there is good evidence for single genes, sometimes with overdominant effects, making major contributions to heterosis [45–47], while heterosis in maize appears to result from reciprocal complementation at a very large number of loci [31, 48]. There must, however, be a role for overdominance in maize, since the continuous purging of deleterious variants in inbred parents through breeding has not decreased heterosis in F_1_ hybrids [25].

In our system, heterosis seems to be driven in a large part by dominant gene expression. This goes in line with the recent finding in rice that non-additive preferred QTLs are the main contributor to heterosis [49]. Both studies also agree that dominant gene actions are more sensitive to environmental factors. It has long been postulated that heterosis is a result of directional dominance [1]. It is therefore noteworthy that we observed a consistent species-wide trend of partial dominance in expression leading to the relative gene-expression level in F_1_ hybrids being more similar to the low-expression parents. With the proviso that we cannot rule out that the absolute transcript abundance of these genes were in fact increasing, our measurements do indicate a relative decrease in cellular transcript concentration, which could have consequences for molecular interactions [50, 51].

We observed putative deleterious effects (i.e., strong deviation of gene expression from the population means) of regulatory rare alleles upstream of both additive and dominant genes, primarily in inbred parental accessions, and to a lesser degree in F_1_ hybrids, suggesting that rare-allele burden has common effects in inbreds and hybrids alike. In addition, in the dominant genes, the abundance of up-stream rare alleles is found to be strongly and negatively associated with heterosis, suggesting that the change of expression, as opposed to being merely a consequence of a common phenotype, had a genetic basis hence potential to respond to selection. Together this indicates that reciprocal complementation in dominant loci is important for heterosis in our system, consistent with the dominance model.

Interestingly, GO enrichment of the heterosis-related dominant genes pointed to repression of reproductive development and abiotic stress response, as well as increased photosynthesis as factors that support more robust growth in F_1_ hybrids. This goes against the perception that genetic complementation is highly genotype-specific hence lacking a common signature. It is possible, therefore, that mildly deleterious alleles are enriched in certain biological functions, likely due to either a small selection coefficient on the resulting trait or conditional neutrality. Another possible explanation of functionally-enriched non-additivity is that factors other than genetic complementation exist upstream of the dominant genes. Two additional lines of evidence suggested the likelihood of this being partially true: for one, enrichment of binding motifs for a small number of transcription factors upstream of the dominant genes raises the possibility of concerted rewiring of transcriptional networks in *trans*; for another, inter-parental genetic distance calculated using neither whole-genome common SNPs nor a subset of those in additive and dominant genes showed a strong correlation with F_1_ growth (Additional file 1: Fig. S15, “Methods” section). Genetic complementation alone, therefore, cannot fully account for heterosis in our system.

Another study recently reported high-parent expression of hub genes from regulatory networks of photosynthesis and cell cycles during early shoot development to be associated with a high degree of growth advantage in one specific F_1_ hybrid [52]. We similarly found the above-MPV expression of photosynthetic function in the later phase of vegetative growth to be positively correlated with increased growth. Both studies also agree in low-parent gene expression being common in F_1_ hybrids during the later stage of vegetative growth.

In contrast, additive (i.e., near-MPV) expression itself does not correlate with the larger size of F_1_ hybrids, hence probably does not directly contribute to hybrid advantage. The observation is consistent with the notion that additive variance is quickly driven to fixation in fitness traits [33]. Additive expression appears to be an intrinsic property for certain genes, mainly enriched in cell-death and stress-response pathways, in F_1_ hybrids, largely independent of specific parental combinations. We conclude that concerted dominant gene expression, rather than canalization via additive gene expression, is a main driver of growth advantage in *A. thaliana* F_1_ hybrids. Whether tighter control of biotic defense responses capacitates hybrid advantage requires further investigation.

A limitation of our study is that we do not know which genes, or rare alleles are causal for the growth advantage in F_1_ hybrids. This could be addressed by eQTL analysis in a larger dataset. This would enable the comparison of hybrid behavior when the hybrids carry the same or contrasting alleles at the eQTL.

## Conclusions

Our systematic use of RNA-seq enabled the parallel comparison of thousands of gene expression traits, all quantified against the same scale. With this, we defined both “additive” and “dominant” genes by their expression at the population level, largely circumventing idiosyncratic behavior of genes in a specific trio, enhancing our confidence that our observations can be generalized across the species. By associating transcriptome changes and plant growth, we were also able to characterize growth as a high-dimensional phenotype, with under-dominance as the predominant type of gene action associated with growth advantage in F_1_ hybrids. Our work provides another step towards understanding molecular mechanisms and evolutionary forces that lead to dominance complementation of rare regulatory alleles.

## Methods

### Generation of genetic resource

Accessions covering the entire species range were chosen from the *A. thaliana* 1001 Genomes Project [26]. F_1_ hybrids used in this study were generated via random crosses, either by randomly crossing individual accessions that reached the flowering stage at the same time (SHB1) [53] or according to a pre-generated randomized crossing scheme after subjecting seedlings to a saturating (12 weeks) vernalization under 4 °C short-day conditions (SD, 8/16 h photoperiod) to synchronize flowering (SHB2).

### Experimental design

The first experiment (Fig. 1A, “SHB1”) initially included 101 parent-F_1_ trios of altogether 286 distinct genotypes. Single plants were grown following an incomplete randomized block design, with each tray as a block within which each genotype was sown as an adjacent pair. The second experiment (Fig. 3A, “SHB2”) included 40 parent-F_1_ trios. Single plants were grown in triplicates, following a split-block design where each block held 10 parent-F_1_ trios with each row consisting of one trio with duplicates of plants in adjacent pots. The trios within each block and the relative positions of genotypes within each trio were randomized. Plants were subjected to either a mock or an artificial defense hormone treatment (see below). After accounting for germination, survival, and initial filtering of RNA-seq outputs, 82 hybrids and 124 inbred parents in SHB1, and 32 trios in SHB2 remained for downstream analyses.

To minimize circadian bias, sowing for both experiments was scheduled in batches to ensure that harvesting could be finished within a 30-min window at the same hour for several consecutive days. At 21 days after sowing, the healthiest appearing plant of each genotype was used for RNA-seq, to ensure any sampling bias is systematically towards the same direction for both inbreds and hybrids. Meanwhile, rosette size measurements were obtained for the same individual plants for which RNA-seq were performed.

For a list of genotypes analyzed in both experiments, see Additional file 2: Table S1.

### Plant culture, treatment, and sampling

Single plants were grown in a 1:1 mixture of calcined clay media (Diamond Pro, Arlington, TX, USA) and vermiculite (Floragard, Oldenburg, Germany) supplemented with liquid growth media [54]. Plants were not vernalized, to ensure that they remained in the vegetative growth phase. As a proxy of vegetative biomass (ref. [55] and Additional file 1: Fig. S1), the rosette area of 21-day-old plants was measured. The full rosettes grown under 16 °C long-day (LD,16/8 photoperiod) were harvested and flash-frozen at 21-day after germination (DAG).

Defense hormone treatment started 14 days after sowing. An analog of the defense hormone salicylic acid (SA), BTH (acibenzolar-S-methyl, Sigma-Aldrich) was used, with optimal dose and treatment scheme of the defense hormone that had been established in a pilot experiment on multiple accessions (Additional file 1: Fig. S2). Each 10 × 6-pot-tray block was the unit of treatment and plants were treated by topical spraying every other day with either a mock solution (20 mL; ddH_2_O, 0.1% DMSO, 0.006% Silwet) or a BTH solution (20 mL; 100 mM acibenzolar-S-methyl, 0.1% DMSO, 0.006% Silwet), and covered for 1 h with transparent plastic lids after spraying. A total of five treatments were administered. Full rosettes were harvested and flash frozen at 21 DAG.

### Growth analysis

Plant growth was monitored by daily image capture from the top of the trays using the RAPA system [56]. Rosette areas were acquired by automatic image segmentation and counting of green pixels, supplemented with manual curation. The rosette size estimates were then converted from pixel counts to mm^2^ by multiplication with a calibration factor.

### RT-qPCR

To establish defense hormone treatment and dosage, the effect of salicylic acid and BTH application was tested by treating 18 accessions with Mock (ddH_2_O, 0.1% DMSO, 0.006% Silwet), 350 mM SA and 100 mM BTH in 3 replicates, each with duplicated plants for phenotyping and qPCR. After 5 treatments the rosettes were harvested in one set of plants to compare their sizes, while the other set of plants were used for qPCR to compare the effect of 350 mM SA and 100 mM BTH treatments. Specifically, RNA was extracted and reverse transcribed. qPCR was performed using SYBER green (Thermo Scientific Maxima SYBR Green qPCR Master Mix (2x)) and primers for *ACTIN2*, *UBC21*, *PR1*, and *NPR1*. Normalization across plates was performed using the same set of samples featured on all plates. The data were analyzed by calculating ΔΔCq (Additional file 1: Fig. S14).

### RNA-seq

RNA-seq libraries were constructed as described [57], using 750 ng total RNA from full rosettes as input. All libraries, each carrying a unique barcode combination were pooled and sequenced in multiple single-end lanes on an Illumina HiSeq 3000 platform for a target coverage of 5 M reads per sample.

### RNA-seq read mapping and post-processing

FASTQ files from multiple lanes were merged and mapped to the TAIR10 transcriptome using *RSEM* (*bowtie2*) with default parameters. Libraries with more than 8 M mapped reads were subsampled to 8 M with *seqtk* prior to mapping. Transcripts mapped to the chloroplast, mitochondria, rDNA clusters, transposable elements (TEs), and pseudogenes, as well as transcripts with an effective length less than 150 nt, were removed from the raw *RSEM* count file. TPM (transcripts per million) counts were then re-estimated for the rest of the genes. Libraries with fewer than 2 M mapped reads and those identified as extreme outliers following a principal-component analysis (PCA) of whole-transcriptome log_2_ (TPM) values were excluded from further analysis. Gene lists were further filtered for average transcript abundance (trimmed mean of log_2_ (TPM) > 0.3) and coefficient of variance > 0.15.

### Additive gene calling

For SHB1 data, the MPV of each gene was calculated for all complete parent-F_1_ trios by taking the arithmetic mean of the parental log_2_ (TPM). Linear regression was then performed between the corresponding F_1_ expression value and the MPV. For SHB2, a linear-mixed model was used to correct for treatment and batch effects. Genes were filtered for regression coefficient > 0.5 and *R*^2^ > 0.4 for SHB1, and regression coefficient > 0.4 and sigma < 0.6 for SHB2. All thresholds were determined by quantiles. Genes called in both SHB1 and SHB2 were taken as common additive genes.

### Non-additive (dominant) gene calling

With SHB1, a population-wide MPV distribution was established for each gene by calculating arithmetic means of log_2_ (TPM) between all possible pairwise combinations of inbred accessions. A two-sided Kolmogorov–Smirnov test was performed per gene to test if the log_2_ (TPM) from the F_1_ hybrids were drawn from the MPV distribution. Genes with *q* < 0.001 (Benjamin-Hochberg FDR) were considered as genes showing expression dominance.

### Bayesian modeling of dominant expression and plant size

Dominant genes from SHB1 were clustered by K-means, with the optimal K determined by the elbow method. A linear-mixed-model (LMM) spline was fitted using the *lme4* package [58] in R (ref. [59]) for gene expression:

$$\mathrm{GeneExpression}\sim \mathrm{IsHybrid}+\mathrm{Size }+\mathrm{Size}:\mathrm{IsHybrid }+ \left(\mathrm{IsHybrid}|\mathrm{LibraryBatch}\right),$$

in which *GeneExpression* is the z-scaled log_2_TPM, *IsHybrid* is a binary code of the hybrid/inbred identity, *Size* is the z-score of rosette size at sampling. Natural cubic splines were modeled for *Size* and *Size-IsHybrid* interaction. The 95% credible intervals for the parameter estimates were established with 10,000 iterations of Bayesian simulation using the *arm* package [60].

### BTH responsive genes

The effect of BTH treatment on gene expression was identified by LMM:

$$\mathrm{GeneExpression }\sim \mathrm{ Treatment }+\mathrm{IsHybrid }+\mathrm{Treatment}*\mathrm{IsHybrid}+\left(1|\mathrm{PlantBatch}\right).$$

To establish a significant threshold, 10,000 permutations were performed for each gene, and the empirical *p*-value was corrected with Benjamin-Hochberg FDR. Genes with q < 0.001 were kept as BTH-responsive genes (*n* = 8797) and examined for their size-MPH correlation.

### Expression-plant size MPH correlation

Expression-MPH and size-MPH were calculated per trio by calculating the per-gene expression and rosette area difference between F_1_ and the MPV in corresponding treatments and replicates. Size MPH-to-expression MPH regression spline was acquired separately for both treatments. An initial round of K-means clustering was performed on the resulting spline coefficients, with the optimal K determined as the division with the highest Dunn index which allows no more than 25% of the clusters carrying less than 5% of the genes. Resulting clusters were inspected and removed if size and expression MPH do not covary. The remaining genes (*n* = 6371) were re-clustered with the same criteria, and the resulting clusters were manually sorted based on size-expression covariation into 12 general categories (Additional file 4: Table S3, Fig. 4D).

### Size-MPH covariation test

To establish the significance of the size-expression correlation, we performed a Wilcoxon signed-rank test on the genes in each of the 61 clusters (Fig. 4B). For each “none” cluster, each gene within the cluster was used as a data point, and the mean rosette size of 4 plants having the lowest and the highest expression MPH of the gene was calculated. The average rosette size corresponding to the two extremes of expression MPH was then compared with a two-sided Wilcoxon signed-rank test. Likewise, for “positive” and “negative” clusters, one-sided tests were used to test for significant differences between average rosette size corresponding to the two extremes of expression MPH within individual clusters. For “quadratic” clusters, separate one-sided tests were performed comparing the samples with extreme expression MPH against those with median expression MPH. Bonferroni correction was used to control for multiple hypothesis testing. The test revealed that our sorting procedure erred on the conservative side: while the top and bottom deciles were significantly different for 17 clusters assigned to the “none” category, only 6 of the “none” clusters were misassigned as “positive”, and none were misassigned as “negative” (Bonferroni corrected *α* < 0.001, Additional file 4: Table S3). The evidence for truly quadratic correlations was less clear.

### GO enrichment

GO enrichment was performed using the *Agrigo* v2 platform [61], with all gene IDs that passed our initial filtering (*n* = 14,067, TAIR10 annotation) as background against the plant GOslim database. Fisher’s exact test was used, and the enrichment *p*-value was corrected using Yekutieli FDR. The enrichment results were visualized with the built-in DAG drawer of *Agrigo* v2.

### Genetic distance

Pairwise SNP Hamming distances were calculated for parental combinations using *PLINK* v.1.90b (ref. [62]). For whole-genome genetic distance, all biallelic SNPs with minor allele frequency (MAF) > 0.2 were used. For sub-genome genetic distance, SNPs were further subset by the genome coordinates (gene body + 1 kb upstream) of target features using *vcftools* v4.2 (ref. [63]). Pearson correlation coefficients were calculated between rosette size MPH and genetic distance either using all common polymorphisms in the genome, or those within the target features of interest.

### Rare allele analysis

Rare (MAF < 0.05), biallelic SNPs 1 kb upstream of gene features were subset from the SNP annotations of the 1001 Genomes Project [26]. Genotype information at these SNPs was acquired for the accessions used in SHB2, and the sum of these rare SNPs upstream of each gene was calculated per accession. For F_1_ hybrids, the upstream rare-allele count was determined by the mean of the rare-allele counts of both parents.

Samples, separated by inbred parents/F_1_ hybrids and with/without BTH treatment, were ranked for their expression values for each gene within a gene list of interest. For each given rank, a gene-list mean upstream rare-allele count was acquired by averaging across all samples that received the same rank in any of the genes within the gene list. Relationships between gene-list mean upstream rare-allele count and expression rank were examined by LOWESS regression and tested with Wilcoxon signed rank sum test between the top and bottom decile of the expression rank.

Likewise, an average rosette-size MPH for each rank was calculated for the gene list, and the Pearson correlation was acquired between average rosette-size MPH and upstream rare-allele count.

### Motif enrichment and de novo motif finding

Motif enrichment and de novo motif finding were carried out using *HOMER* v4.10.4 (ref. [64]) with TAIR10 reference genome and gene annotation. For every set of candidate genes, genomic sequences 1 kb upstream from the transcription start site (TSS) and 1 kb downstream from the transcription termination site (TTS) were indexed from the strand-specific gene coordinates. Both assays were performed by using the *findMotifsGenome.pl* function and *HOMER*'s in-built plant promoter motif database as reference:

$$findMotifsGenome.pl\,\left[\mathrm{geneset}\_\mathrm{promoter}\_\mathrm{coord}.\mathrm{bed}\,\right] \left[\mathrm{TAIR}10\_\mathrm{customref}\,\right] \left[\mathrm{motif}\_\mathrm{outputdir}\right].$$

$$-size\;given-\mathrm S\;15-preparse-mcheck\,\left[\mathrm{homer}\_\mathrm{database}/\mathrm{data}/\mathrm{knownTFs}/\mathrm{plant}/\mathrm{all}.\mathrm{motifs}\right].$$

The top known motif hit (in all cases *p* ≤ 10^–10^) from each candidate set was then used for a second motif enrichment step, where the promoters and downstream sequences were searched for the significant motif using *HOMER*’s *annotatePeaks.pl* function:

$$annotatePeaks.pl\,\left[\mathrm{geneset}\_\mathrm{promoter}\_\mathrm{downstream}.\mathrm{bed}\right] \,\left[\mathrm{TAIR}10\_\mathrm{customref}\right]$$

$$-m\,\left[\mathrm{motif}\_\mathrm{outputdir}\right]/\mathrm{knownResults}/\mathrm{known}1.\mathrm{motif }-size given -nmotifs -mbed$$

$$\left[\mathrm{output}1.\mathrm{bed}\right]>\left[\mathrm{output}1.\mathrm{txt}\right],$$

to generate corresponding genomic coordinates, and subsequently associated back to the genes containing the motif of interest in their regulatory regions (*bedtools* v2.26.0 intersect) [65].

### Supplementary Information

**Additional file 1: Figure S1.** Rosette area serves as a good predictor of rosette biomass. **Figure S2.** Linear-model-based additive gene calling. **Figure S3.** Examples showing that non-additive genes exhibit various forms of dominance in F_1_s. **Figure S4.** BTH treatment reduced rosette size in both inbreds and F_1_s. **Figure S5.** Randomly selected genes do not show differential expression-size correlation in inbreds and hybrids. **Figure S6.** Rosette size in inbred parental lines and F_1_ hybrids. **Figure S7.** Consistent and significant reduction in rosette area by BTH in both batches of the SHB2 experiment. **Figure S8.** Reaction norm of rosette area (mm^2^ ) after mock and BTH treatments for all inbred parents and F_1_ hybrid trios. **Figure S9.** BTH-responsive genes sorted into 61 clusters. **Figure S10.** Positive genes are enriched for genes encoding thykaloid-localized proteins that are involved in photosynthetic process. **Figure S11.** Top 3 motif enrichment results for All-BTH negative genes. **Figure S12.** Common additive genes. **Figure S13.** Additive genes in SHB2. **Figure S14.** Efficient induction of defense responses in *A. thaliana* accessions with the BTH dosage used. **Figure S15.** Genetic distance correlates poorly with absolute rosette size mid-parent heterosis.

**Additional file 2: Table S1.** Sequenced and analyzed genotypes from SHB1 and SHB2.

**Additional file 3: Table S2.** Dominant genes called in SHB1, and their cluster identity.

**Additional file 4: Table S3.** Wilcoxon rank sum test on the size-expression MPH spline clusters.

**Additional file 5: Table S4.** Genes negatively associated with heterosis under BTH or mock + BTH conditions (“negative genes”).

**Additional file 6: Table S5.** Genes positively associated with heterosis under both mock and BTH conditions (“positive genes”).

**Additional file 7: Table S6.** 300 common additive genes.

**Additional file 8: Table S7.** Whole-genome SNP hamming distance for the parental accessions included in the study.

**Additional file 9. **Review history.

## Data Availability

Raw sequencing data is available at the ENA under the accession ERA9420648 [66] and ERA9420737 [67]. Code to generate the results and the gene expression matrix are available at: https://github.com/weigelworld/SigHeterosis [68] under the MIT Open Access License. Archival versions are available under the MIT Open Access License at: https://zenodo.org/record/8249142 [69].
