# Supplementary material for: Pervasive under-dominance in gene expression underlying emergent growth trajectories in Arabidopsis thaliana hybrids
Source: Genome Biol. 2023 Sep 4;24:200. doi: 10.1186/s13059-023-03043-3 (PMC10478501; doi:10.1186/s13059-023-03043-3)
Supplement: Supplementary file 1 — Additional file 1: Figure S1. Rosette area serves as a good predictor of rosette biomass. Figure S2. Linear-model-based additive gene calling. Figure S3. Examples showing that non-additive genes exhibit various forms of dominance in F1s. Figure S4. BTH treatment reduced rosette size in both inbreds and F1s. Figure S5. Randomly selected genes do not show differential expression-size correlation in inbreds and hybrids. Figure S6. Rosette size in inbred parental lines and F1 hybrids. Figure S7. Consistent and significant reduction in rosette area by BTH in both batches of the SHB2 experiment. Figure S8. Reaction norm of rosette area (mm2 ) after mock and BTH treatments for all inbred parents and F1 hybrid trios. Figure S9. BTH-responsive genes sorted into 61 clusters. Figure S10. Positive genes are enriched for genes encoding thykaloid-localized proteins that are involved in photosynthetic process. Figure S11. Top 3 motif enrichment results for All-BTH negative genes. Figure S12. Common additive genes. Figure S13. Additive genes in SHB2. Figure S14. Efficient induction of defense responses in A. thaliana accessions with the BTH dosage used. Figure S15. Genetic distance correlates poorly with absolute rosette size mid-parent heterosis. [file 13059_2023_3043_MOESM1_ESM.pdf]

Supplementary Figures

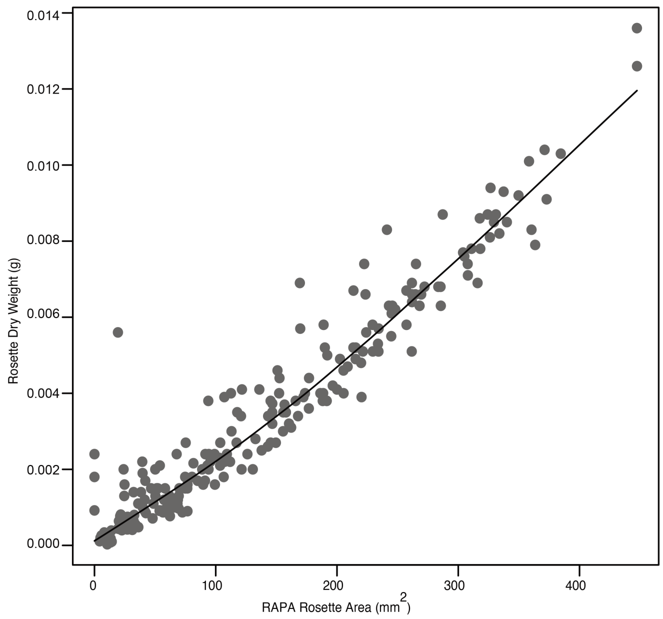

Figure S1. Rosette area serves as a good predictor of rosette biomass. Pearson correlation coefficient (R= 0.96, p<2.2e-16) of biomass (g) with rosette area (mm^2) as measured on the RAPA system at 21 days after sowing of 221 individuals of mixed genotypes (grey dots), and LOWESS trendline.

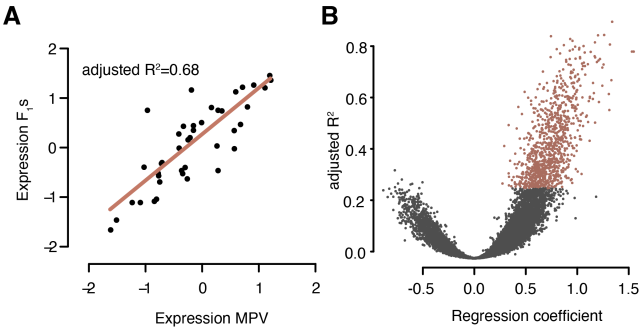

Figure S2. Linear-model-based additive gene calling. A. One representative additive gene (At5G43740, a CC-NBS-LRR class disease resistance protein, dominance rank: 16,574) whose expression in F_1 hybrids closely correlates with calculated mid-parent value, both showed as z-score. The red line shows the regression line. B. Volcano plot of R^2 value of each gene plotted against the corresponding linear-regression coefficient. Red dots show genes that passed the filtering threshold.

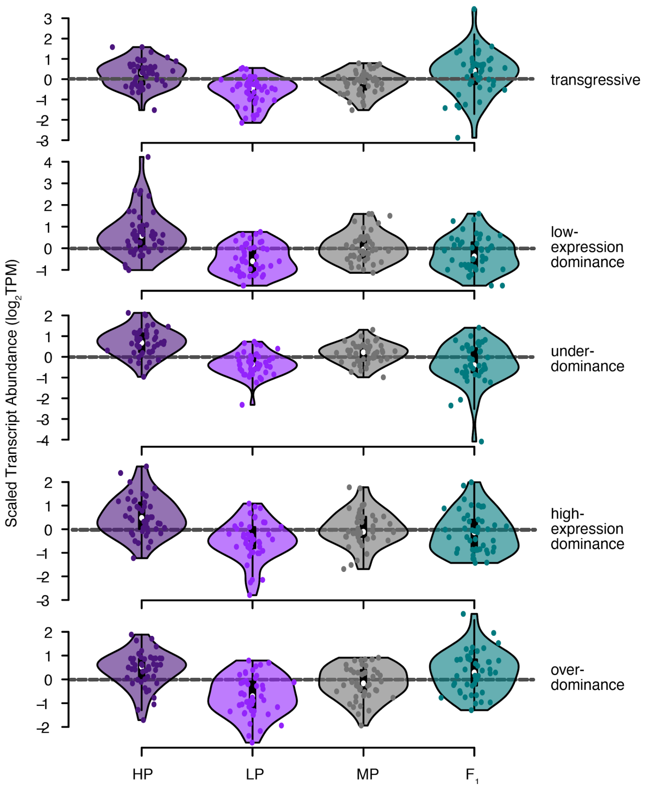

Figure S3. Examples showing that non-additive genes exhibit various forms of dominance in F_1s. From top: AT5G07960, AT3G61170, AT2G17670, AT2G27510, AT4G12970.

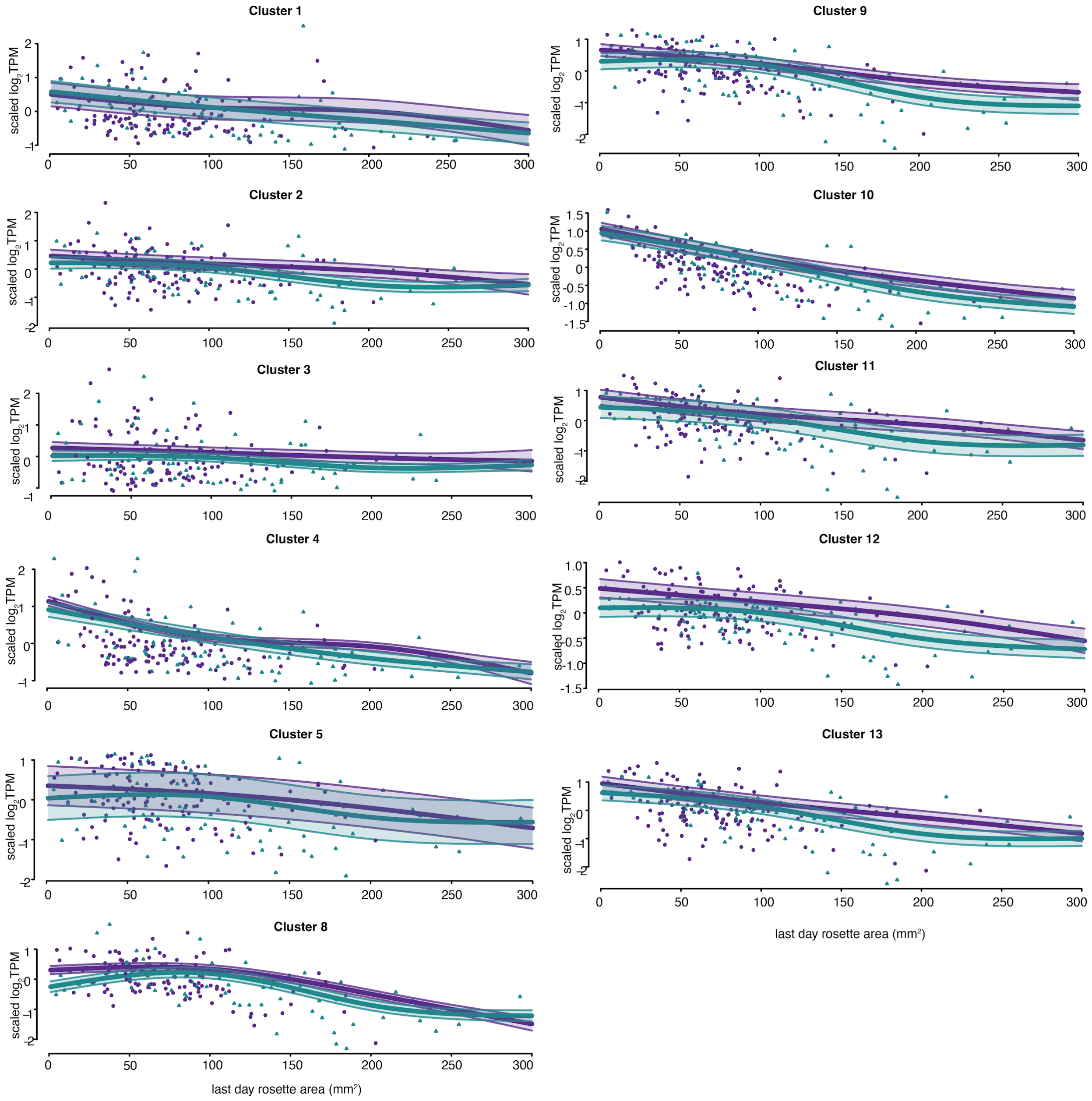

Figure S4. BTH treatment reduced rosette size in both inbreds and F_1s. LMM spline fitting of cluster mean expression levels with 95% Bayesian credible intervals for dominant gene clusters not shown in Fig. 3. Mean expression level across all genes within a cluster for inbred parents (purple dots) and F_1 hybrids (turquoise dots) against last day rosette area (mm^2) were plotted. Clusters 1-5 showed little rosette size-expression level association, while clusters 8-13 showed monotonic decrease of cluster mean expression level with increased plant size. While F_1 hybrids exhibited the same trend as the inbred parents, the mean expression levels are consistently lower in F_1 hybrids across the entire rosette size range for cluster 8-13.

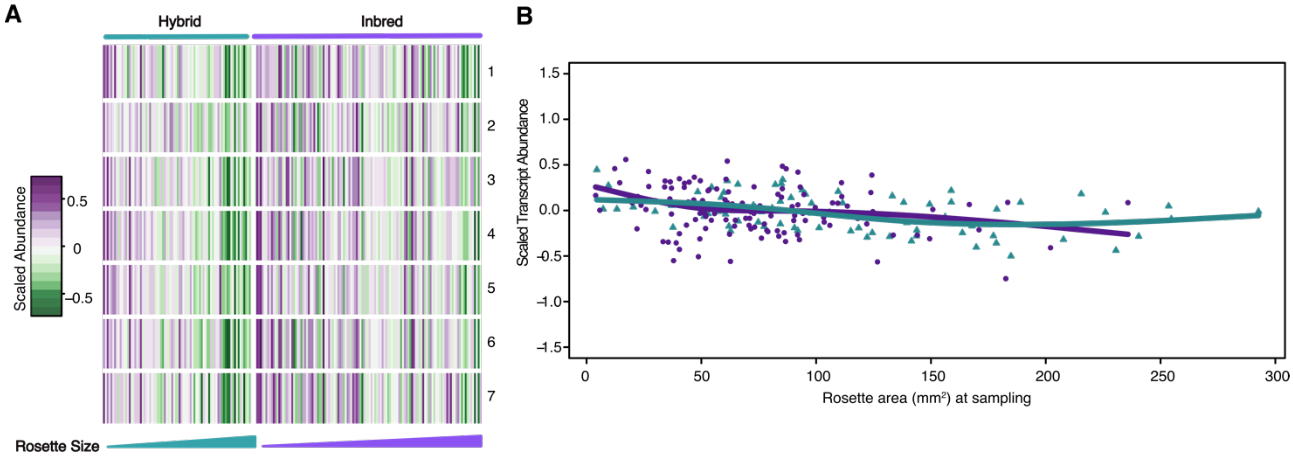

Figure S5. Randomly selected genes do not show differential expression-size correlation in inbreds and hybrids. A. Heatmap of the average expression of gene clusters from 500 randomly sampled genes, arranged in the same order as in Fig. 2A. B. Randomly sampled genes, which show little expression-size covariation and no differences between inbreds and F_1s.

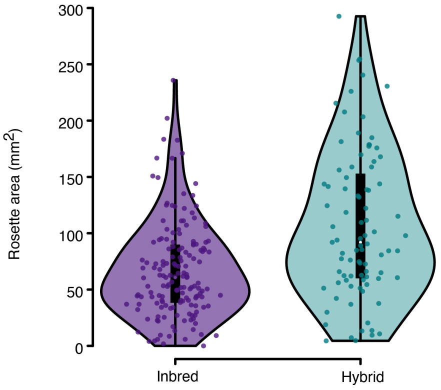

Figure S6. Rosette size in inbred parental lines and F_1 hybrids. The F_1 population has significantly more large individuals than the inbred population ($p=2x10^{-5}$, Two-tailed Kolmogorov-Smirnov). F_1s: 107.1±66.1 mm^2, n=82; parents: 67.2±42.5 mm^2, n=124. In a comparison of randomly chosen F_1s and inbreds, the F_1 hybrid was twice as likely than the inbred to be the larger individual (Cliff's delta=0.33).

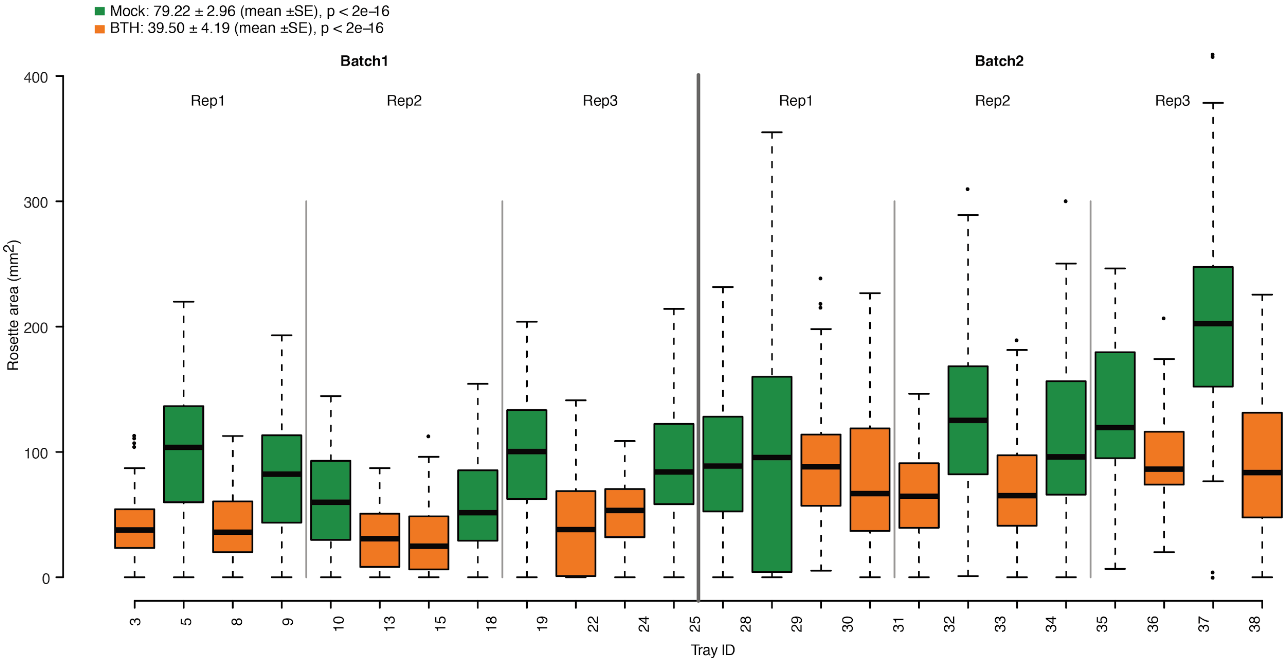

Figure S7. Consistent and significant reduction in rosette area by BTH in both batches of the SHB2 experiment. Each tray was a treatment unit. Shown are boxplots of rosette area (mm^2) in each tray with mock (green) or BTH treatment (orange). Only trays with parent-hybrid trios further used for transcriptome analysis are included. The rosette area after treatment with BTH was reduced compared to mock treatment (2-way nested ANOVA, mock: 79.2 ± 3.0 mm^2, BTH treatment: 39.5 ± 4.2 mm^2; $p < 10^{-16}$).

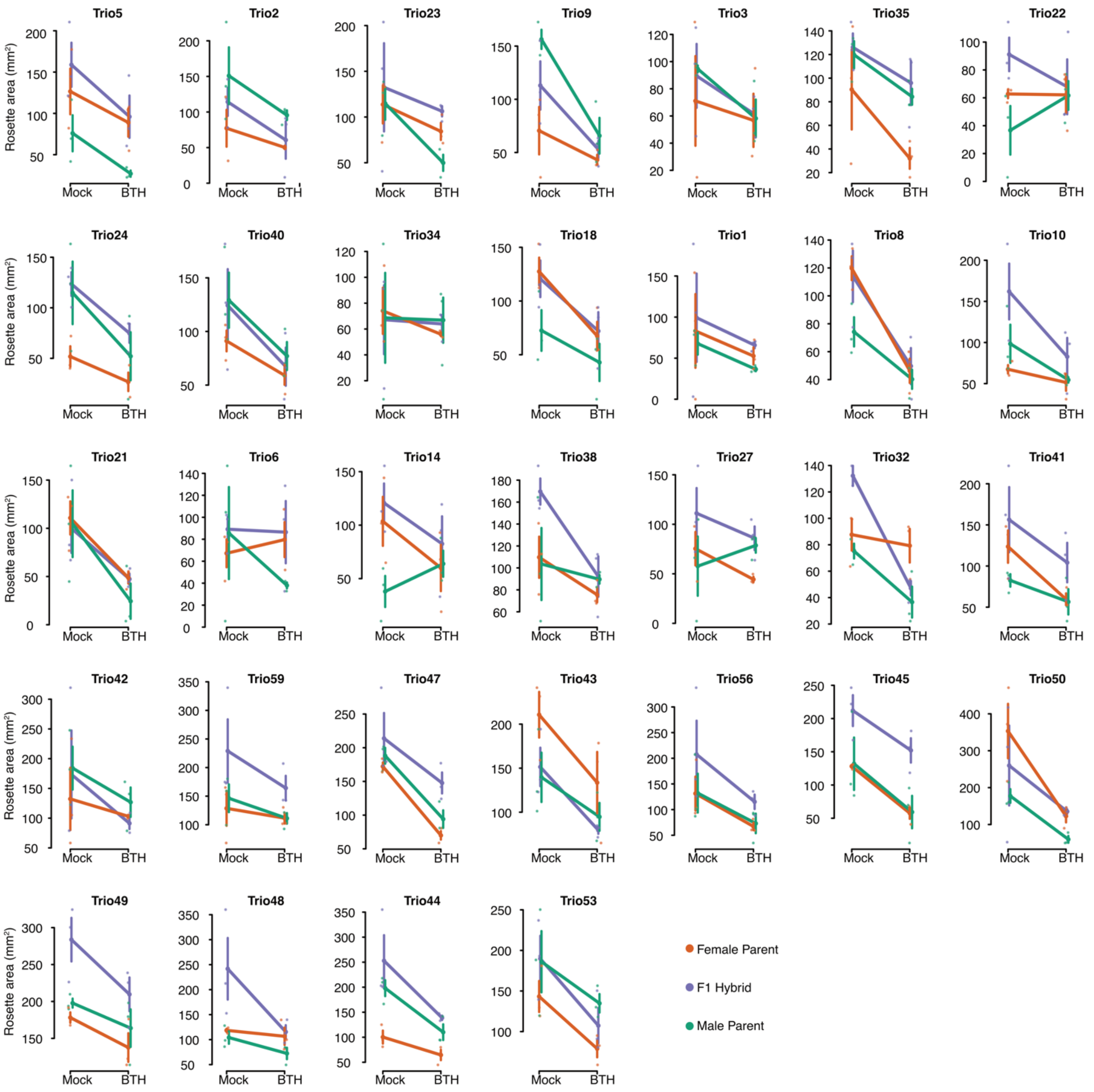

Figure S8. Reaction norm of rosette area (mm^2) after mock and BTH treatments for all inbred parents and F$_1$ hybrid trios. Solid lines connected the mean rosette area under the two treatments, with the dots illustrating individual rosette area of each plant and the error bars showing standard deviation of the biological replicates.

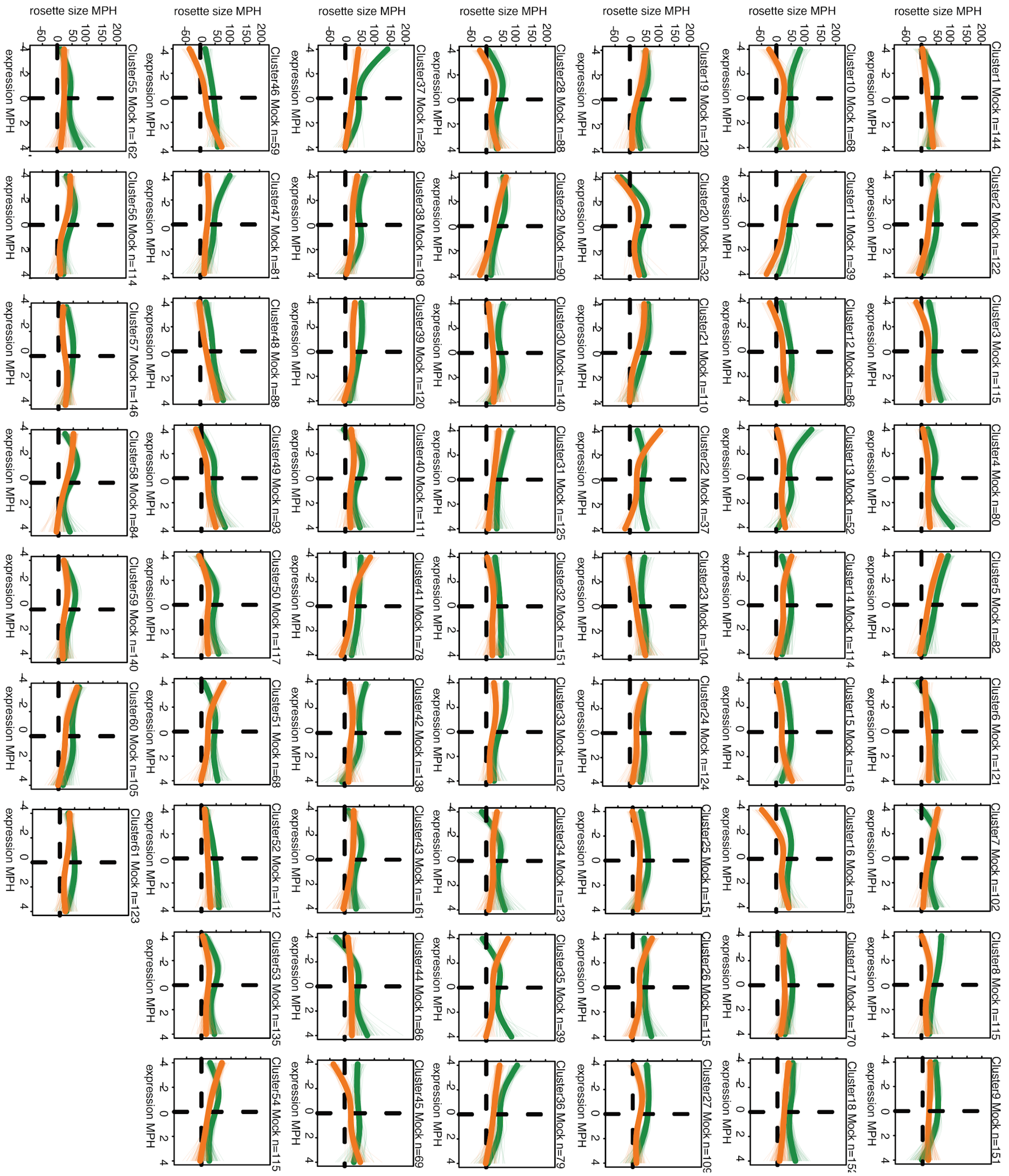

Figure. S9. BTH-responsive genes sorted into 61 clusters. Genes were sorted based on spline regression of rosette area MPH (mm^2) to their expression MPH across all trios. Shown are a graphic representation of the clusters. Each thin line represents a gene, and the thick line represents cluster mean (green: mock, orange: BTH). The 61 clusters were subsequently sorted into 12 general categories based on the regression trends in mock and BTH treatments.

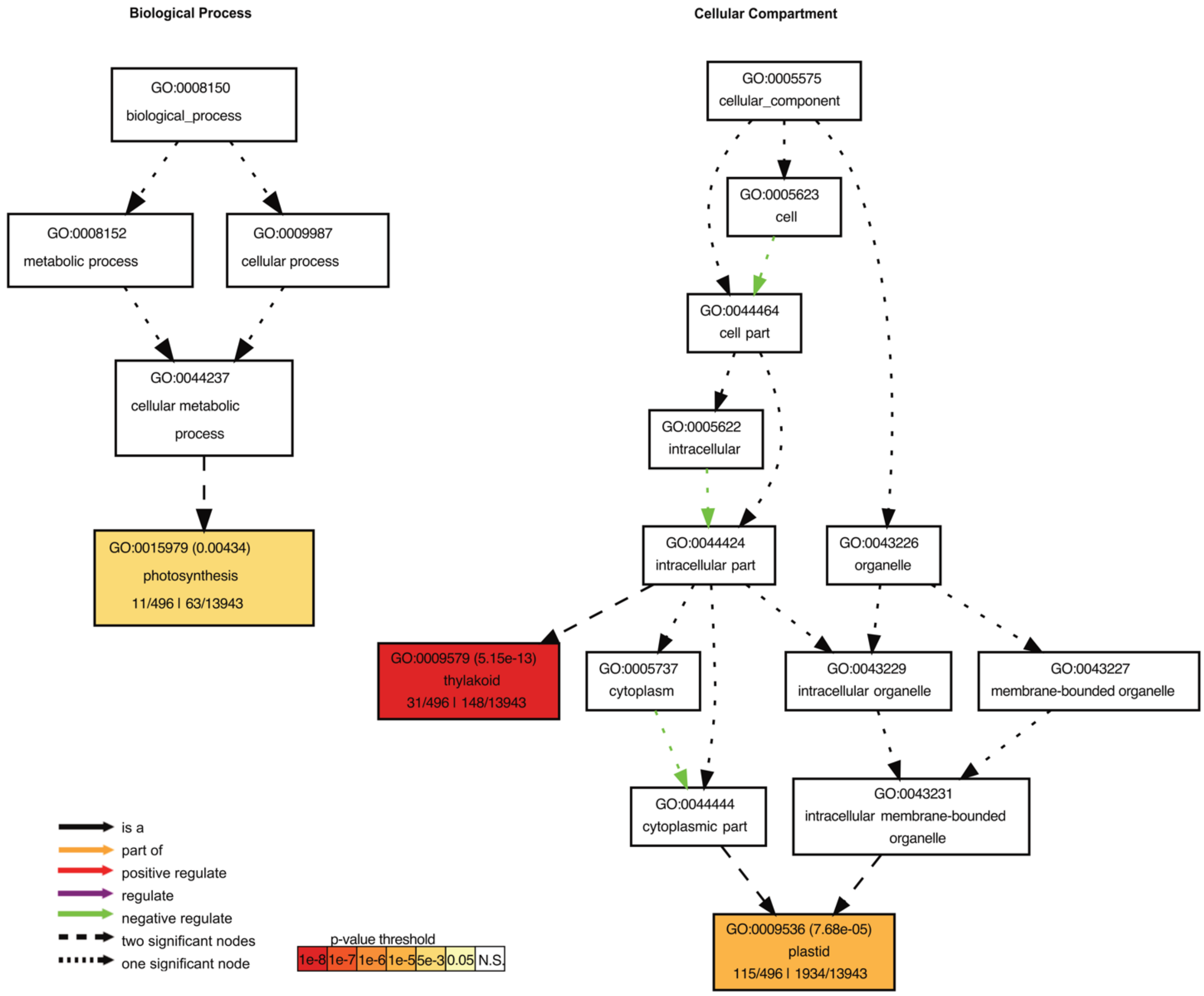

Figure S10. Positive genes are enriched for genes encoding thykaloid-localized proteins that are involved in photosynthetic process. Fisher's Exact Test was used against the background list of filtered expressed genes in SHB2 dataset, using plant GOslim based on TAIR10 annotation.

Rank	Motif	Name	P-value	% Target	No. Target	% Bkgd	No. Bkgd
1	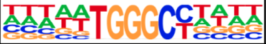	PCF	1e-42	26.65	599	15.27	7252
2	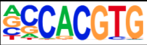	c-Myc	1e-21	19.26	433	12.13	5761
3	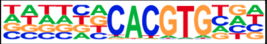	BZR	1e-21	16.55	372	9.99	4743

Figure S11. Top 3 motif enrichment results for All-BTH negative genes. All-BTH negative genes are all genes showing negative correlations between dominance of expression with dominance in F1 rosette size when treated with BTH). No.Target: number of genes from All-BTH negative gene list carrying target motif in *cis*, No.Bkgd: number of genes from background gene list carrying target motif in *cis*.

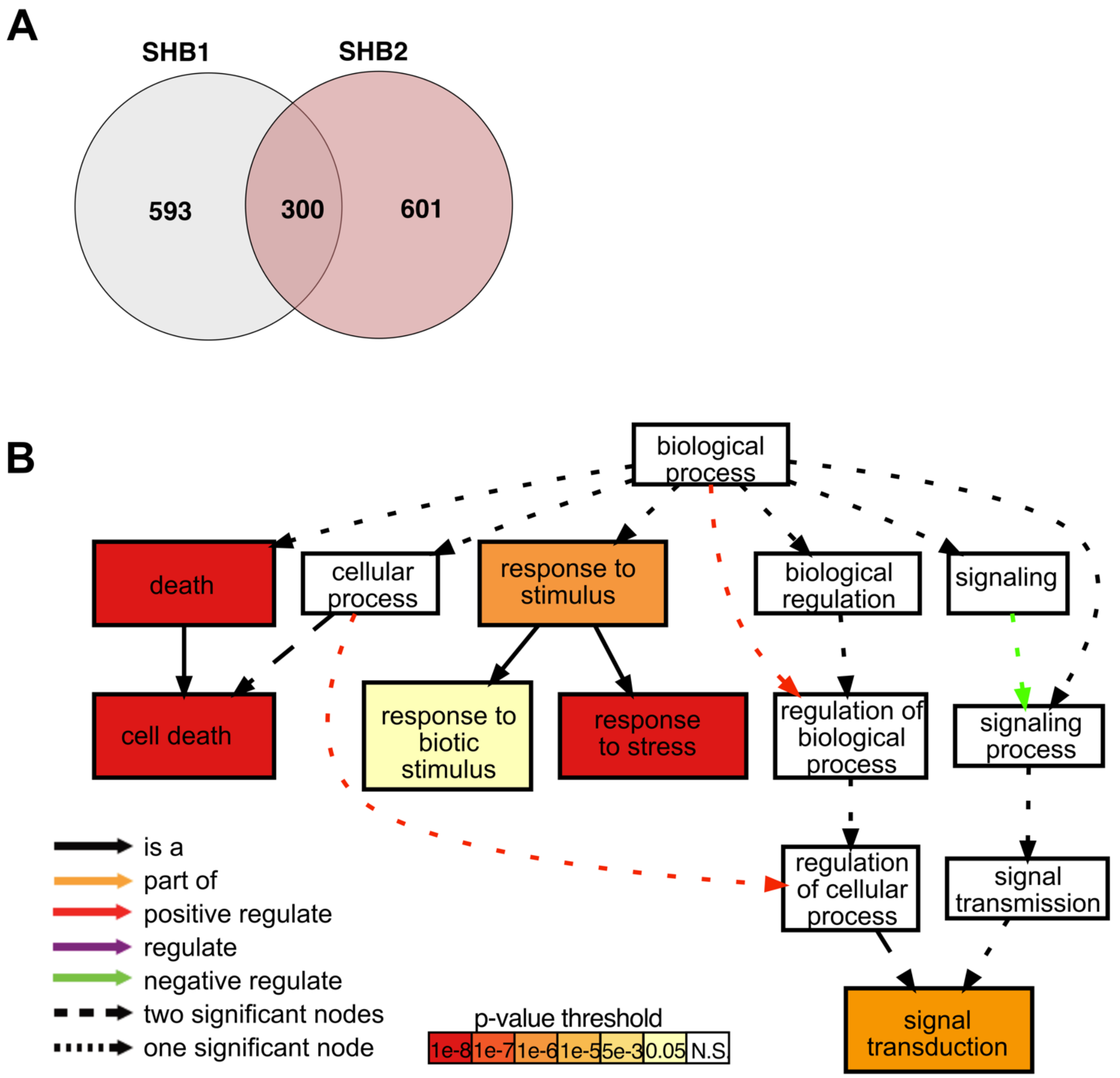

Figure S12. Common additive genes. A. Venn diagram showing the overlapping of additive genes in SHB1 and SHB2 experiments. B. GO-term enrichment diagram of common additive genes.

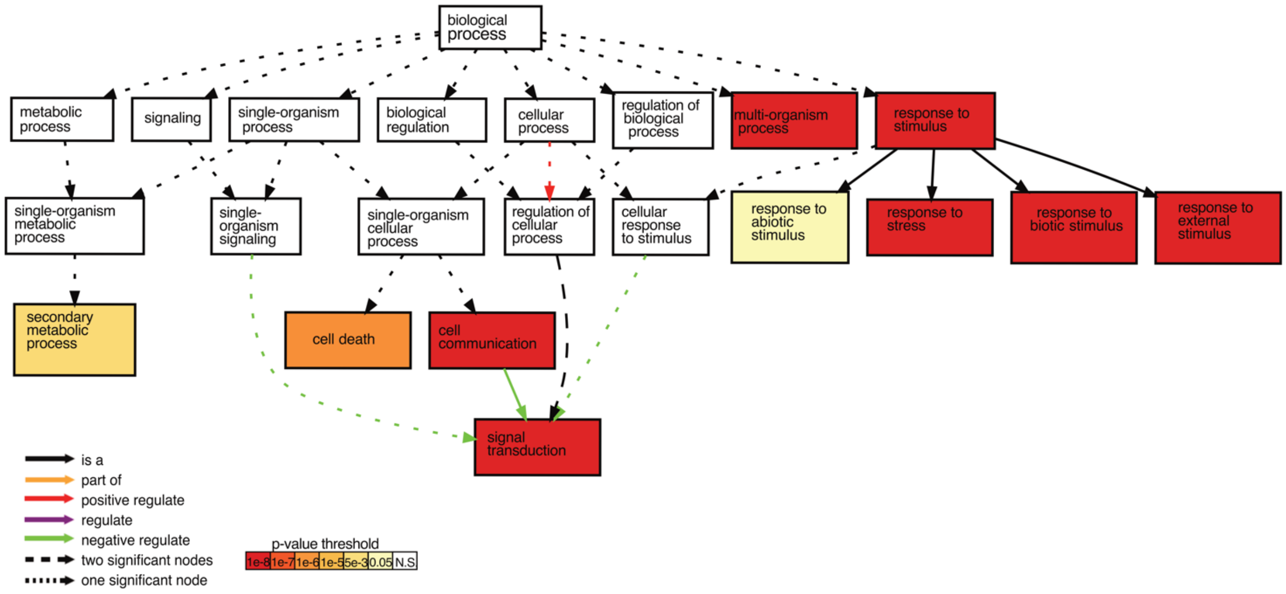

Figure S13. Additive genes in SHB2. This gene set (n=901) is enriched for GO terms of response to stress and (biotic) stimuli, cell death, and secondary metabolic process.

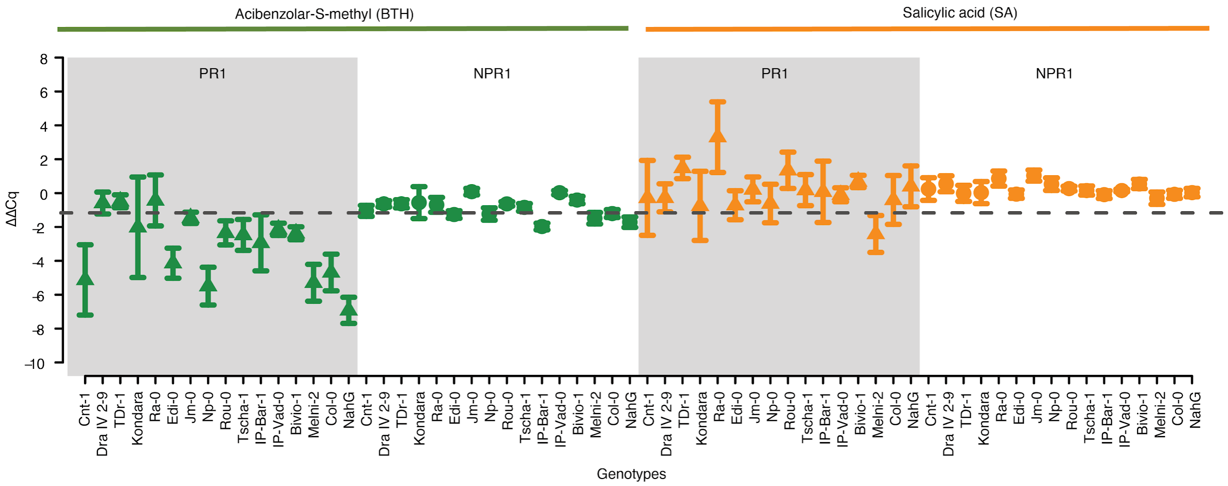

Fig. S14. Efficient induction of defense responses in *A. thaliana* accessions with the BTH dosage used. qRT-PCR of two defense marker genes, *PR1* and *NPR1*, in 16 inbred accessions after BTH and SA treatment. Each dot represents the mean ΔΔCq value against housekeeping genes in mock-treated plants, with error bars indicating the standard deviation of the biological replicates.

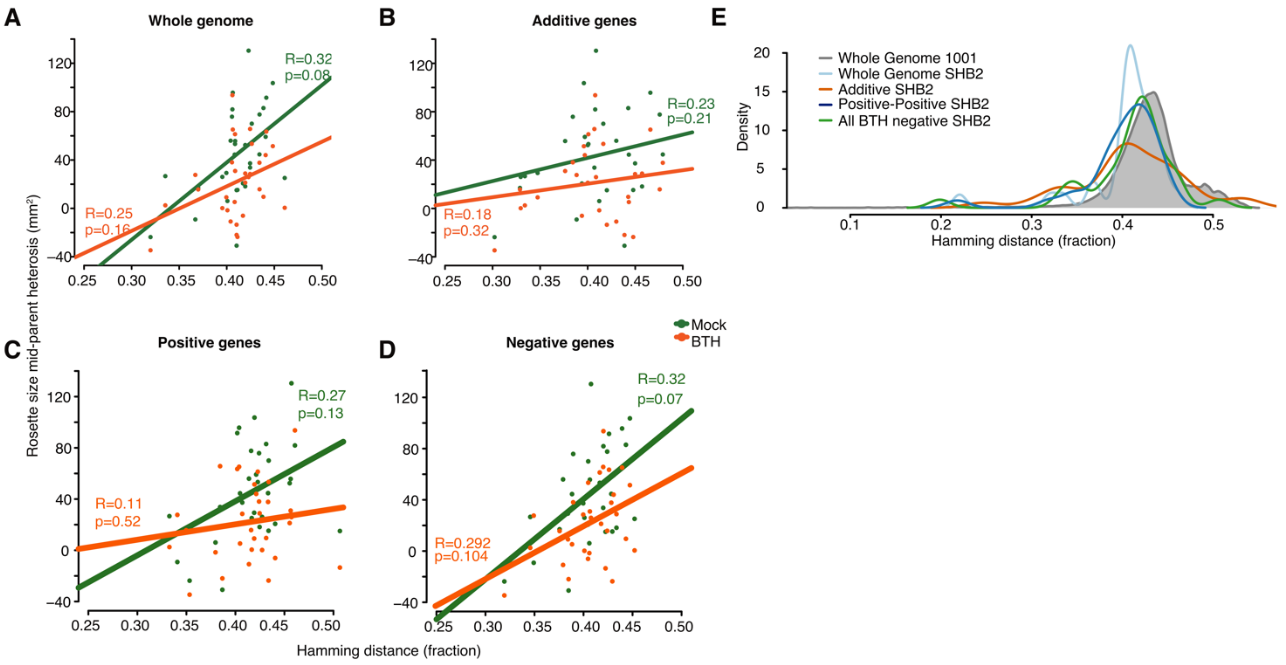

Fig. S15. Genetic distance correlates poorly with absolute rosette size mid-parent heterosis. A-D. Absolute rosette size mid-parent heterosis (mm^2) regressed against inter-parental hamming distance (as fractions of total polymorphic SNPs) calculated using whole genome SNPs, or SNPs located within the B. additive genes, C. positive genes, and D. Negative genes. E. Kernel density plots of inter-parental hamming distance as the fraction of all filtered diallelic SNPs. Subsetting SNPs to given gene context did not dramatically change the hamming distance distribution. Curve in shaded grey shows the whole-genome hamming distance profile of all 1001 genome accessions.
