# Supplementary material for: Pervasive under-dominance in gene expression underlying emergent growth trajectories in Arabidopsis thaliana hybrids
Source: Genome Biol. 2023 Sep 4;24:200. doi: 10.1186/s13059-023-03043-3 (PMC10478501; doi:10.1186/s13059-023-03043-3)
Supplement: Supplementary file 9 — Additional file 9. Review history. [file 13059_2023_3043_MOESM9_ESM.docx]

Review History

**First round of review**

**Reviewer 1**

**Were you able to assess all statistics in the manuscript, including the appropriateness of statistical tests used?**

Yes, and I have assessed the statistics in my report.

**Comments to author:**

In this manuscript, the authors systematically studied the differential expression patterns of genes among F1s and parents in 141 randomly crossed Arabidopsis thaliana, where heterosis is widespread in biomass and defense responses phenotypes. Among the genes expressed in both F1s and parents, ~900 additive and 1805 non-additive genes were identified. In Arabidopsis, most of the latter (non-additive genes) were expressed lower than the median parental value (incomplete recessive), and the pattern may be distinct from that in hybrid crops. Generally, the insights presented in this study by using population-level transcriptome data are very important and can server as references for exploring the molecular mechanisms of heterosis in more plants.

I only have three minor questions/suggestions for the work:

1. Line 383, F1 growth. 1 should be subscript.

2. The population-scale expression profiling is useful for identifying trans- or cis- eQTLs. Is there any discussion on this topic?

3. Besides the raw reads and codes, the authors should provide the gene expression matrix, the genotype matrix and related results as well. I notice Supplementary Data Sets only provide the information of gene lists.

Xuehui Huang

**Reviewer 2**

**Were you able to assess all statistics in the manuscript, including the appropriateness of statistical tests used?**

Yes, and I have assessed the statistics in my report.

**Comments to author:**

The authors examine the relationship between dominance in gene expression and growth in Arabidopsis F1 hybrids. They find >1,000 genes that show some type of dominance effect. They study how these genes with dominance effects relate to plant growth and the rare allele burden of an individual.

Overall, I found the paper an interesting study of how the phenomenon of dominance relates to gene expression and rare genetic variants at a population level. I think this is rigorous study. I have some suggestions that are intended to help with clarity.

Comments:

- Throughout the terms non-additivity and dominance are used in a somewhat interchangeable way. This is not technically correct, as dominance is just one contributor to non-additivity (the other main one is epistasis). I think the language around the relationship between non-additivity and dominance, and how this relates to analyses and experimental design could be more precise throughout the paper. E.g., on p1, the authors write 'Additive and non-additive genetic effects are best revealed in heterozygotes, in which contributions of different alleles to a phenotype of interest can be easily partitioned'. This experimental design is good for dominance but can in fact be more challenging for studying other sources of non-additivity.

-Around lines 97-100, the total number of genes that were included in the analysis should also be mentioned.

-Lines 111-113, 'Because expression level close to parental average would be indicative of canalization'. This seemed like a strong assertion that comes out of nowhere and I was not sure how well supported this statement is. Perhaps soften the statement using instead of 'could' or provide references supporting this logic.

-Figure 2, this figure took a while to understand, potentially because of aesthetic choices. I wondered if the figure could be simplified. 2C and D could be moved to the supplement. 2C in particular is a bit confusing as it looks like most of the rows in 2A. When I first looked at 2A, the feature that most popped to me was Cluster 7, the one that seems more muted in its magnitude. It was only after going back and reading the text that I understood the point here, which is that Cluster 6 is the interesting one.

-Figures 4A and B, the labels 'Rosette size nonadditivity (mm2)' was a bit unclear and should be explained in the legend. Is this the rosette size corrected for the additive expectation?

-Figure 4D, the network is hard to read. There is a different presentation in the supplement with the network split into a couple pieces. Maybe the version in the supplement should be moved into the main text.

-Figure 5D-F, I was not sure what the mean rare allele count represents. I read the Methods but this seems like the count is being divided by a number to create a measure that goes from 0-1.0. A little more information in the Methods and legend would help.

-Figure 5G-I, same comment as earlier about 'Rosette size nonadditivity (mm2)'.

**Authors Response**

**Point-by-point responses to the reviewers’ comments:**

Reviewer 1

In this manuscript, the authors systematically studied the differential expression patterns of genes among F1s and parents in 141 randomly crossed Arabidopsis thaliana, where heterosis is widespread in biomass and defense responses phenotypes. Among the genes expressed in both F1s and parents, ~900 additive and 1805 non-additive genes were identified. In Arabidopsis, most of the latter (non-additive genes) were expressed lower than the median parental value (incomplete recessive), and the pattern may be distinct from that in hybrid crops. Generally, the insights presented in this study by using population-level transcriptome data are very important and can server as references for exploring the molecular mechanisms of heterosis in more plants.

I only have three minor questions/suggestions for the work:

1. Line 383, F1 growth. 1 should be subscript.

*-->We corrected this, and performed another round of proof reading to fix other typos.*

2. The population-scale expression profiling is useful for identifying trans- or cis- eQTLs. Is there any discussion on this topic?

*-->In our dataset, our power to detect eQTLs is limited. We have incorporated in our discussion what a larger dataset would enable.*

3. Besides the raw reads and codes, the authors should provide the gene expression matrix, the genotype matrix and related results as well. I notice Supplementary Data Sets only provide the information of gene lists.

*-->The gene expression matrix in the form of TPM tables for both experiments is available through the institutional GitHub(https://github.com/weigelworld/SigHeterosis), as well as the genotype matrix in the form of whole-genome hamming distance estimated using SNP calls from the 1001 Genomes.*

Reviewer 2

The authors examine the relationship between dominance in gene expression and growth in Arabidopsis F1 hybrids. They find >1,000 genes that show some type of dominance effect. They study how these genes with dominance effects relate to plant growth and the rare allele burden of an individual.

Overall, I found the paper an interesting study of how the phenomenon of dominance relates to gene expression and rare genetic variants at a population level. I think this is rigorous study. I have some suggestions that are intended to help with clarity.

Comments:

- Throughout the terms non-additivity and dominance are used in a somewhat interchangeable way. This is not technically correct, as dominance is just one contributor to non-additivity (the other main one is epistasis). I think the language around the relationship between non-additivity and dominance, and how this relates to analyses and experimental design could be more precise throughout the paper. E.g., on p1, the authors write 'Additive and non-additive genetic effects are best revealed in heterozygotes, in which contributions of different alleles to a phenotype of interest can be easily partitioned'. This experimental design is good for dominance but can in fact be more challenging for studying other sources of non-additivity.

*--> We agree that the experimental setup is good for dominance but hard for studying epistasis. We have made it clearer in the manuscript that there are two major sources of non-additivity, and our focus is on dominance. The word “non-additive” in the manuscript has also been replaced with “dominant”, unless when discussing the general phenomenon of non-additivity.*

-Around lines 97-100, the total number of genes that were included in the analysis should also be mentioned.

*-->Total number of gene added. The sentence now reads: “Out of 16,667 expressed genes that passed our filter (Materials and Methods), we identified close to 900 genes that were consistently expressed in an additive fashion (Fig. S2, Methods), and 1,805 genes (Data S2) that were consistently expressed in a dominant fashion”.*

-Lines 111-113, 'Because expression level close to parental average would be indicative of canalization'. This seemed like a strong assertion that comes out of nowhere and I was not sure how well supported this statement is. Perhaps soften the statement using instead of 'could' or provide references supporting this logic.

*-->We rephrased the sentence to “If a gene is typically expressed close to the parental average, then the expression values in the parents will be more broadly distributed than in the hybrids, because extreme parents would usually have been crossed to less extreme parents. We therefore interpret our result as indicating that…”*

-Figure 2, this figure took a while to understand, potentially because of aesthetic choices. I wondered if the figure could be simplified. 2C and D could be moved to the supplement. 2C in particular is a bit confusing as it looks like most of the rows in 2A. When I first looked at 2A, the feature that most popped to me was Cluster 7, the one that seems more muted in its magnitude. It was only after going back and reading the text that I understood the point here, which is that Cluster 6 is the interesting one.

*--> We moved Figure 2C-D to the supplement as the new Figure S5. We also added more description in the figure legend, that the dominant genes showed three types of association between their expression levels and rosette size: no association (cluster 1-5), positive association (cluster 6), and negative association (cluster 7-13). Most importantly, for both positive- and negative- associations, the systematic differences in expression levels across the entire rosette-size range seen in F1 hybrids are consistent with F1s being larger than parents.*

-Figures 4A and B, the labels 'Rosette size nonadditivity (mm2)' was a bit unclear and should be explained in the legend. Is this the rosette size corrected for the additive expectation?

*--> The understanding is correct. The Y-axis labels are changed to rosette size MPH (mm2). In addition, the following text was added to the legend: “B. Average biomass MPH (the amount that F1 rosette size differs from that of corresponding parental mean) for rosette samples…”*

-Figure 4D, the network is hard to read. There is a different presentation in the supplement with the network split into a couple pieces. Maybe the version in the supplement should be moved into the main text.

*--> Did as suggested. Thank you.*

-Figure 5D-F, I was not sure what the mean rare allele count represents. I read the Methods but this seems like the count is being divided by a number to create a measure that goes from 0-1.0. A little more information in the Methods and legend would help.

*--> The mean rare allele count in the hybrid was not divided by any normalizer, and the small value observed is simply a result of the alleles being rare. The following texts are added to Figure 5 legend: “Average upstream rare allele counts were calculated sensu Kremling et al. 2018 [31]: For each gene within the gene list (the cluster), all inbred samples received a rank based on their expression value. Across the gene list, average upstream rare allele counts of all samples sharing the same rank were plotted as points, and lines indicate LOWESS trend lines… F1 samples are ranked by expression value the same way as the inbreds in A-C. The rare allele count for the F1s is calculated as the average number of rare alleles between their corresponding parents.”*

-Figure 5G-I, same comment as earlier about 'Rosette size nonadditivity (mm2)'.

*See above.*

**Second round of review**

**Reviewer 2**

I think this looks nice. My only comment regards the sentence, which should be modified:

'Additive and non-additive, especially dominant genetic effects are best revealed in heterozygotes, in which contributions of different alleles to a phenotype of interest can be easily partitioned.'

The wording is a bit confusing and also I think this statement is inaccurate as worded. Additive and epistatic alleles are most easily identified in RILs or haploids, not heterozygotes. However, dominant alleles can only be seen in heterozygotes. Thus, heterozygotes must be used to study dominance.

**Authors Response**

**Point-by-point responses to the reviewers’ comments:**

Reviewer #2:

I think this looks nice. My only comment regards the sentence, which should be modified:

'Additive and non-additive, especially dominant genetic effects are best revealed in heterozygotes, in which contributions of different alleles to a phenotype of interest can be easily partitioned.'

The wording is a bit confusing and also I think this statement is inaccurate as worded. Additive and epistatic alleles are most easily identified in RILs or haploids, not heterozygotes. However, dominant alleles can only be seen in heterozygotes. Thus, heterozygotes must be used to study dominance.

*--> Thank you for helping with clarification. We rephrased the sentence to: "Hybrids provide a unique opportunity to study the dominant portion of non-additive effects, enabling direct comparison of the relative importance of additivity and dominance to phenotypes of interest".*
